# A RIAM/lamellipodin–talin–integrin complex forms the tip of sticky fingers that guide cell migration

**DOI:** 10.1038/ncomms9492

**Published:** 2015-09-30

**Authors:** Frederic Lagarrigue, Praju Vikas Anekal, Ho-Sup Lee, Alexia I. Bachir, Jailal N. Ablack, Alan F. Horwitz, Mark H. Ginsberg

**Affiliations:** 1 Department of Medicine, University of California San Diego, La Jolla, California 92093, USA; 2 Department of Cell Biology, University of Virginia, Charlottesville, Virginia 22908, USA

## Abstract

The leading edge of migrating cells contains rapidly translocating activated integrins associated with growing actin filaments that form ‘sticky fingers’ to sense extracellular matrix and guide cell migration. Here we utilized indirect bimolecular fluorescence complementation to visualize a molecular complex containing a Mig-10/RIAM/lamellipodin (MRL) protein (Rap1-GTP-interacting adaptor molecule (RIAM) or lamellipodin), talin and activated integrins in living cells. This complex localizes at the tips of growing actin filaments in lamellipodial and filopodial protrusions, thus corresponding to the tips of the ‘sticky fingers.’ Formation of the complex requires talin to form a bridge between the MRL protein and the integrins. Moreover, disruption of the MRL protein–integrin–talin (MIT) complex markedly impairs cell protrusion. These data reveal the molecular basis of the formation of ‘sticky fingers’ at the leading edge of migrating cells and show that an MIT complex drives these protrusions.

## Abstract (web summary)

The leading edge of migrating cells contains activated integrins associated with growing actin filaments that form ‘sticky fingers’ to guide cell migration. Here, the authors detect a complex of MRL proteins, talin and activated integrins in lamellipodia and filopodia in living cells, comprising the tips of the ‘sticky fingers’.

## Graphical abstract

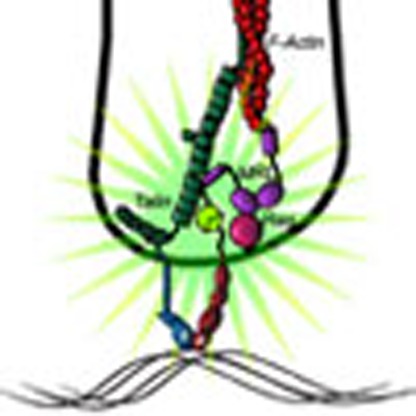

Cell migration is crucial to diverse processes such as embryonic development, tissue repair, axon extension/path finding and cancer metastasis. Cells migrating in a mesenchymal mode form actin-driven protrusions, such as lamellipodia and filopodia, at the leading edge^1^,^2^. These protrusions help to maintain cell polarity and the directional persistence of cell migration^3^,^4^,^5^,^6^

Integrins are sensors of the chemical and physical nature of the extracellular matrix and cells can dynamically increase the affinity of integrins for their ligands, which is operationally defined as integrin activation^7^. Activated integrins are enriched at the leading edge of migrating cells^8^ where they help to direct migration^9^. A potential connection between integrin activation and actin dynamics in directional migration was revealed by studies showing that activated integrins are associated with polymerizing actin filaments and move transversely in the lamellipodium and along filopodia^10^. Galbraith *et al*.^10^ termed these structures as ‘sticky fingers’ and proposed that they played a role in directional cell migration and path finding however, the molecular basis of these structures and their functional role remained unclear.

The binding of talins to the β subunit cytoplasmic tail is a final common step in β_1_, β_2_ and β_3_ integrin activation *in vitro*^11^ and *in vivo*^7^. In purified systems, talin binding is sufficient to activate integrins^12^. Once activated, integrins engage immobilized ligands, such as components of the extracellular matrix, to form adhesions, a constellation of integrin-driven assemblies of structural and signalling components that dictate many cellular behaviours^13^. The entire spectrum of β_1_, β_2_ and β_3_ integrin-based adhesions contains talin^14^ and talin is required for adhesion formation^13^.

Ras GTPases^15^, in particular, Ras subfamily members, Rap1a and Rap1b, stimulate integrin activation^16^ and several Rap1 effectors have been implicated in integrin activation^15^,^17^,^18^. Rap1-GTP-interacting adaptor molecule (RIAM) is a Rap1 effector that is a member of the Mig-10/RIAM/lamellipodin (MRL) family of adaptor proteins^17^,^19^,^20^ that mediates the invasion and migration of melanoma cells. RIAM contains Ras association and pleckstrin homology domains and proline-rich regions, which are defining features of the MRL protein family. Lamellipodin (Lpd) is a RIAM paralogue that is also present in many cells^20^ and plays an important role in direction finding during cell migration *in vivo* and *in vitro*^21^. MIG-10, the *Caenorhabditis elegans* orthologue, is known to be involved in axonal path finding^19^. Previously, we exploited the fact that agonists fail to activate recombinant α_IIb_β_3_ expressed in CHO (chinese hamster ovary) cells to develop a synthetic reconstruction of an integrin activation pathway and used it in combination with forward and reverse genetics to dissect the pathway^22^. We found that Rap1-induced formation of a complex containing the Rap1 effector, RIAM and talin, which results in talin recruitment to the plasma membrane and to integrin α_IIb_β_3_; this complex represents an early modular component of integrin-based adhesions formed by one of several mechanisms that drive the integrin–talin interaction^23^. Mapping studies identified short amphipathic helices in RIAM or Lpd that bind talin; joining those helical peptides to the membrane targeting sequences of Rap1 led to a minimized Rap-MRL module that was sufficient to recruit talin to activate integrins^24^. Thus, MRL proteins function as a scaffold that in effect connects the membrane targeting sequences in Ras GTPases to talin, thereby recruiting talin to the plasma membrane and activating integrins.

Because RIAM or Lpd can drive the formation of an integrin–talin complex containing activated integrins^24^ and because both paralogues are enriched at the protruding leading edge of migrating cells^17^,^20^, we hypothesized that an MRL protein–integrin–talin (MIT) complex forms the tip of the ‘sticky fingers’ in migrating cells. Here we develop and validate an indirect bimolecular fluorescence complementation (BiFC) method to visualize the MIT complex in living cells. We find that the MIT complex is enriched at the tips of growing actin filaments in lamellipodial and filopodial protrusions corresponding to the tips of ‘sticky fingers’. Formation of the complex requires that talin bridges integrin and MRL proteins. Moreover, disruption of the MIT complex using a RIAM mutant defective in talin binding results in impaired cell protrusion. Thus, we reveal the molecular basis of formation of ‘sticky fingers ‘ at the leading edge of migrating cells.

## Results

### An MIT complex is at the tips of sticky fingers

**Figure 1:**
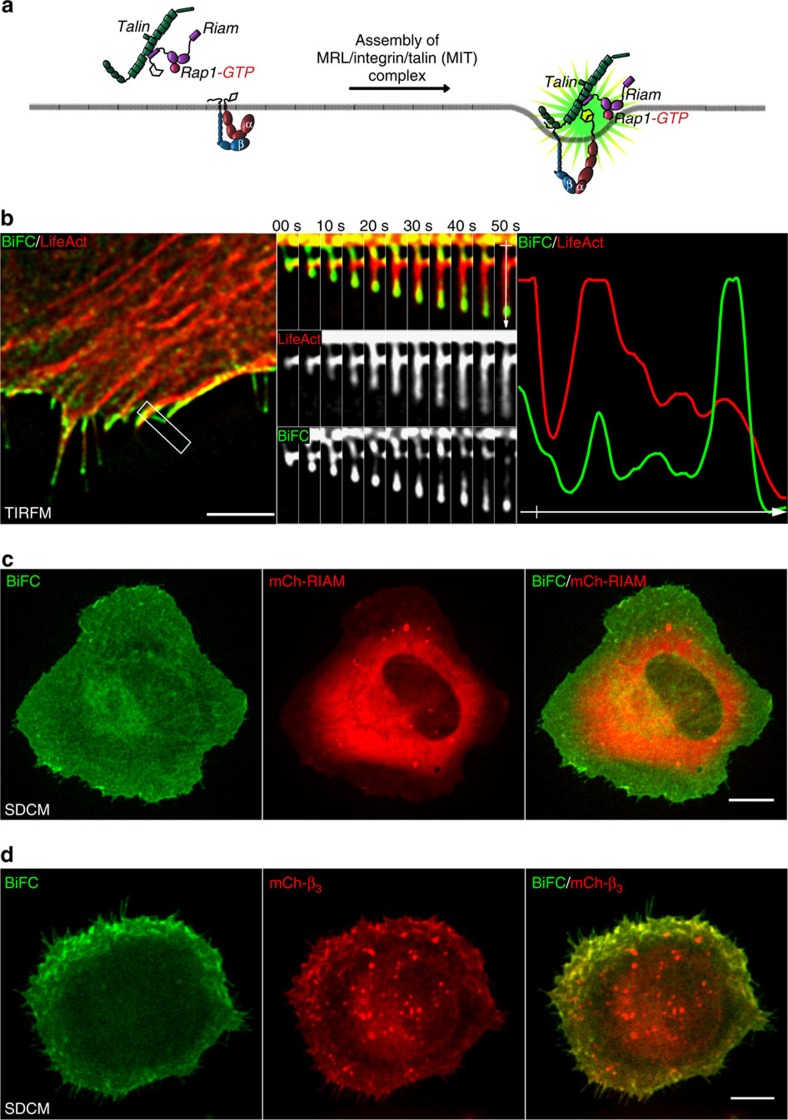
The RIAM/integrin/talin complex is at the tips of growing actin filaments. (**a**) Principle of BiFC imaging of the RIAM/integrin/talin complex. The N-terminal moiety of Venus (VN) is joined to the N terminus of RIAM. The C-terminal moiety of Venus (VC) is joined to the cytoplasmic tail of the integrin α subunit. When Rap1–RIAM–talin interacts with the integrin, the Venus N- and C-terminal moieties are brought into proximity and refold to form a functional fluorescent Venus molecule. Therefore, the presence of a fluorescent signal will report the presence of RIAM/integrin/talin complex. (**b**) U2-OS cells expressing VN-RIAM, integrin α_IIb_-VCβ_3_, and mCherry-LifeAct were plated on fibrinogen for 2 h and imaged with time-lapse TIRFM. A segment of the cell edge is enlarged and displayed as a movie montage at 5 s intervals. ImageJ software was used to perform a line scan from the base of the filopodium to its tip. In TIRFM images *t*he filopodial tips sometimes lift off the substrate and are no longer visible in the TIRF plane. Scale bar, 5 μm. (**c**,**d**) U2-OS cells expressing VN-RIAM, integrin α_IIb_-VCβ_3_, and either mCherry-RIAM (**c**) or mCherry-β_3_ (**d**) were plated on fibrinogen for 2 h and imaged with spinning disk confocal microscopy (SDCM). Note the absence of BiFC co-localizing with the cytosolic pool of RIAM and the integrin β_3_-containing vesicles. Scale bar, 10 μm.

Active integrins can localize to the tips of actin-based protrusions to form ‘sticky fingers’ that probe the extracellular matrix^10^. Activated Rap1 causes RIAM to bind to talin, resulting in the association of talin with integrins and consequent integrin activation^24^,^25^,^26^. We used BiFC^27^,^28^ to specifically visualize the complex of RIAM with talin bound to integrins (Fig. 1a). The N-terminal β-sheet moiety of the Venus (VN) was joined to the N-terminus of RIAM and the C-terminal β-sheet moiety of the Venus (VC) was attached to the cytoplasmic tail of the integrin α-subunit. We reasoned that, since the interaction of RIAM with the integrin requires an endogenous talin bridge^23^,^24^, the presence of BiFC would reveal the complex of RIAM/talin/integrin (Fig. 1a). U2-OS cells expressing VN-RIAM and α_IIb_-VCβ_3_ in combination with Lifeact (to visualize filamentous actin^29^ in live cells) exhibited BiFC at the tips of growing actin filaments within lamellipodia and filopodia (Fig. 1b; Supplementary Movies 1 and 2). To exclude potential path length effects in this striking enrichment of BiFC, we examined its distribution in comparison with markers for bulk cytoplasm (blue fluorescent protein (BFP)) or membrane (BFP-Ras-CAAX). Line scans along the filopodia from the base to their tips demonstrated that the BiFC signal was selectively enriched at the tip (Supplementary Fig. 1a,b). Similarly, when we performed this experiment in NIH-3T3 (Supplementary Movie 3) or Ptk1 (Supplementary Movies 4 and 5) cells which form large lamellipodia, we observed localization of BiFC at the edge of protruding lamellipodia, particularly at the tips of actin filaments traversing the lamellipodia. Thus the RIAM/integrin/talin complex is present at the tips of growing actin filaments that either traverse lamellipodia or drive filopodial extension.

We also observed co-localization of BiFC with Myosin-X, an established marker of filopodia^30^ at the tips of filopodia (Supplementary Fig. 2a). Furthermore, we compared the BiFC signal versus the overall abundance of RIAM and β_3_ integrin. We observed that BiFC was prominent at the plasma membrane in protrusions, but was neither associated with the bulk of cytoplasmic RIAM nor with intracellular vesicles containing integrin β_3_, indicating that BiFC represented only a fraction of total cellular RIAM and integrin (Fig. 1c and d; Supplementary Fig. 1c and d).

### BiFC depends on the interaction of talin with the integrin

**Figure 2:**
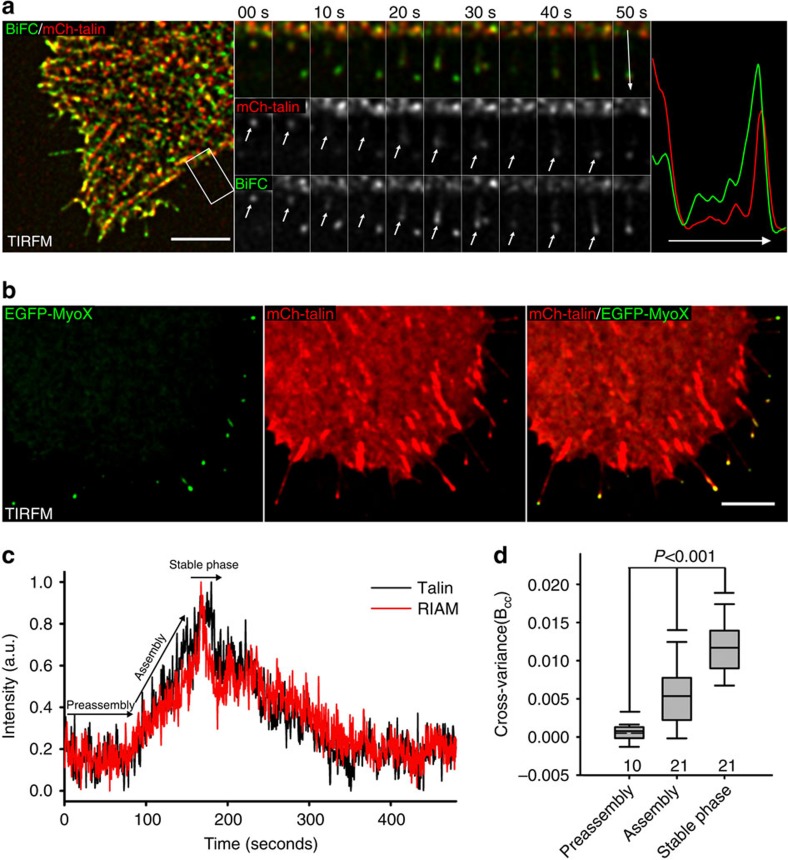
BiFC co-localizes with talin. (**a**) mCherry-talin and BiFC co-localized at the tip of the filopodium. U2-OS cells expressing VN-RIAM, integrin α_IIb_-VCβ_3_ and mCherry-talin were plated on fibrinogen for 2 h before imaging with time-lapse TIRFM. A segment of the cell edge is enlarged and displayed as a movie montage at 5 s intervals. The arrows show the BiFC enrichment at the tip of the growing filopodium. The line scan was drawn along the extended filopodium as highlighted in the box at 50 s. Scale bar, 5 μm. (**b**) U2-OS cells expressing EGFP-Myosin-X and mCherry-talin were plated on fibronectin for 2 h before imaging with TIRFM. Scale bar, 5 μm. (**c**,**d**) RIAM associates with talin in molecular complexes during formation of nascent adhesion. CHOK1 cells expressing talin1-mGFP and RIAM-mCherry were plated on cover slips coated with 2 μg ml^−1^ fibronectin and imaged with time-lapse TIRFM every 0.5 s for 8 min. (**c**) Fluorescence intensity traces of talin and RIAM in a selected nascent adhesion that forms and disassembles as the cell edge protrudes. (**d**) Cross-variance parameter (*B*_cc_) calculated from 30 s intensity time segments corresponding to different stages of nascent adhesion formation. Whiskers show the 5th and 95th percentiles, lower and upper box edges show the 25th and 75th percentiles respectively; central solid and dotted lines are the median and mean respectively. We used ANOVA analysis on ranks for multiple distributions and Mann–Whitney test for two-way comparisons. Time segments were sampled from 12 adhesions resulting in 10 preassembly, 21 assembly and 20 stationary time segments data points.

As noted above (Fig. 1a), BiFC between RIAM and the integrin should require a talin bridge. In support of this idea, we observed that talin was usually present when BiFC was present in protrusive structures (Fig. 2a; Supplementary Fig. 3a; Supplementary Movie 6), although the vast majority of talin was either cytoplasmic or present in focal adhesions. As noted above, Myosin-X, an authentic marker of filopodial tips, was co-localized with BiFC. Similarly, in cells co-transfected with enhanced green fluorescent protein (EGFP)-Myosin-X and mCherry-talin, there was clear localization of a minor fraction of talin in the Myosin-X-tagged filopodial tips (Fig. 2b) in addition to the expected prominent focal adhesion localization. Importantly, talin is prominent in vinculin-containing mature focal adhesions^14^ but BiFC was much reduced in these more mature focal adhesions (Supplementary Fig. 3b,c; Supplementary Movie 7), consistent with the previous finding that vinculin inhibits RIAM binding to talin^23^,^31^, thereby displacing RIAM from adhesions. Conversely, even though vinculin can induce talin association with and activation of integrins^23^, vinculin was not evident in BiFC-positive structures in protrusions (Supplementary Fig. 3b,c; Supplementary Movie 7).

To determine when RIAM and talin reside in a common complex, we used cross-variance analysis^32^,^33^. This method detects the presence of molecular complexes using the covariance of fluorescence fluctuations arising from dynamic exchange of molecules in adhesions. In CHOK1 cells, talin-mGFP and RIAM-mCherry localize in nascent adhesions as the cell edge protrudes (Fig. 2c). We observed positive cross-variance between talin and RIAM as the adhesion starts to assemble and persists even after the adhesion forms (Fig. 2d). Thus, RIAM and talin reside in a common molecular complex as nascent adhesion form^32^,^33^.

**Figure 3:**
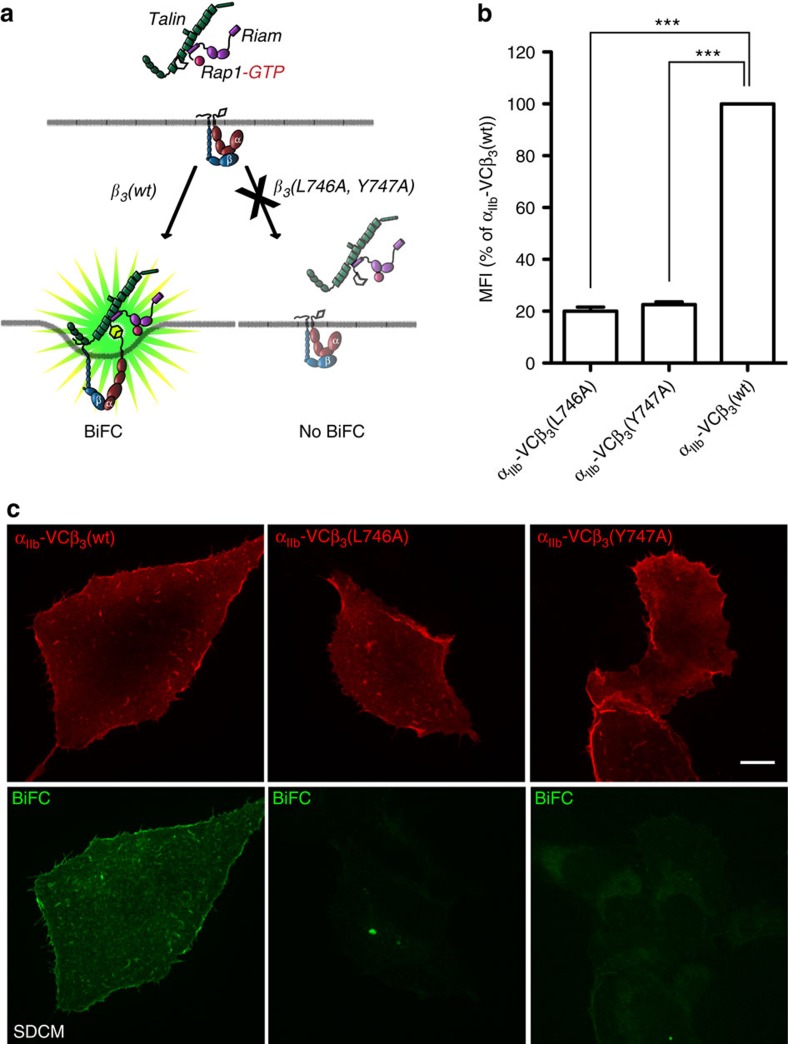
Formation of the MIT complex depends on talin interaction with the integrin. (**a**) Predicted effects of blocking talin’s interactions on BiFC. Mutations of integrin β_3_(L746A) and β_3_(Y747A) interfere with talin binding. (**b**) U2-OS cells expressing VN-RIAM were infected with lentiviral particles encoding α_IIb_-VCβ_3_, α_IIb_-VCβ_3_(L746A) or α_IIb_-VCβ_3_(Y747A). Cells were harvested, fixed, stained with anti-α_IIb_β_3_ (D57) antibody and BiFC fluorescence was measured by flow cytometry. The mean of BiFC fluorescence intensity of the D57-positive population was plotted. Data are expressed as percentage of cells expressing α_IIb_-VCβ_3_(WT). One-way analysis of variance with Bonferroni’s test were used to compare the populations: *** *P*<0.001. The error bars display s.e.m. for four separate experiments. (**c**) U2-OS cells expressing VN-RIAM were transduced as described in **b** were plated on fibronectin for 2 h. The cells were fixed, stained with anti-α_IIb_β_3_ (D57) antibody and imaged for BiFC by spinning disc confocal microscopy. Scale bar, 10 μm.

To directly test whether BiFC required talin–integrin interaction, we introduced the β_3_(Y747A) mutation that inhibits the binding of talin, filamin and other cytoplasmic proteins or the β_3_(L746A) mutation that selectively reduces integrin interactions only with talin^11^ (Fig. 3a). In cells expressing either α_IIb_-VCβ_3_(L746A) or α_IIb_-VCβ_3_(Y747A) there was a dramatic reduction in the BiFC signal as assayed by quantitative flow cytometry (Fig. 3b) and by spinning disk confocal microscopy (Fig. 3c). Thus, we conclude that BiFC reports the presence of an MIT complex.

### The MIT complex is regulated by Rap1

**Figure 4:**
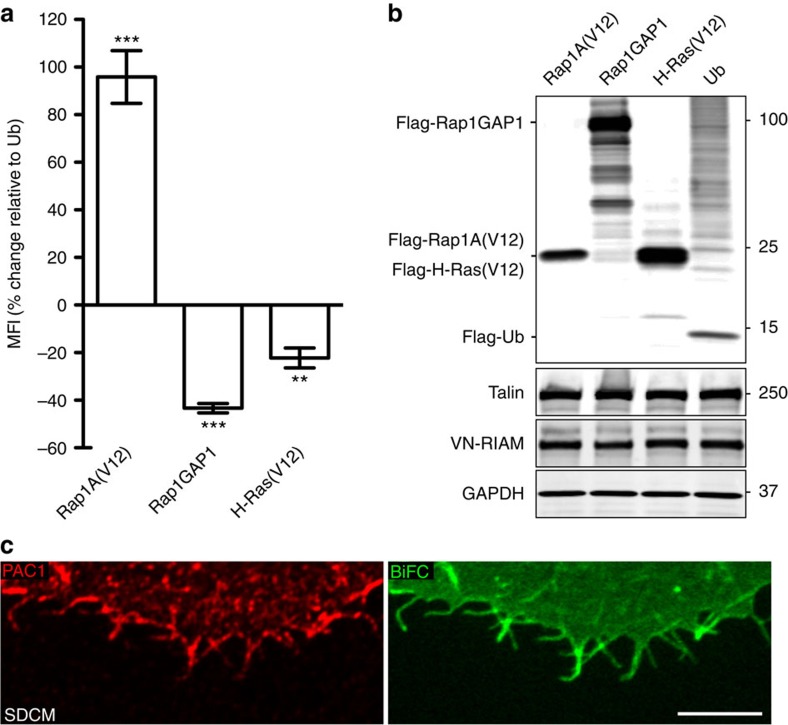
The RIAM–MIT complex is regulated by Rap1 and is associated with activated integrins. (**a**) U2-OS cell clone stably expressing integrin α_IIb_-VCβ_3_ with inducible expression of VN-RIAM were transiently transfected with constructs encoding 3xFlag-tagged Rap1(V12), Rap1GAP1, H-Ras(V12) or ubiquitin. Twenty-four hours later, expression of VN-RIAM was induced with doxycycline. See Supplementary Fig. 4 for characterization of the U2-OS Tet-On cell line with tunable BiFC. Cells were harvested, fixed, permeabilized and stained with anti-Flag antibody and BiFC fluorescence intensity was analysed by flow cytometry. The mean BiFC fluorescence intensity of the Flag-positive population was plotted as relative to the BiFC fluorescence of cells expressing ubiquitin as a control. One-way analysis of variance with Bonferroni’s test was used to compare each condition versus ubiquitin: **P<0.01; ***P<0.001. Error bars display s.e.m. for four separate experiments. (**b**) Lysates of cells described in **a** were analysed by Western blot with anti-Flag, anti-talin and polyclonal anti-VN antibodies. GAPDH was used as a loading control. (**c**) U2-OS cells expressing VN-RIAM and integrin α_IIb_-VCβ_3_ were plated on fibrinogen for 2 h. The cells were incubated with PAC1 antibody before being fixed, stained with secondary antibody, and imaged by spinning disk confocal microscopy. Scale bar, 10 μm.

We exploited the observation that the activity of Rap1 (refs 24, 25) regulates the co-precipitation of RIAM with talin and integrins to test whether BiFC reported the physiological regulation of the MIT complex in living cells. Suppression of endogenous Rap1 activity by expression of Rap1GAP1 markedly reduced the BiFC signal as assayed by quantitative flow cytometry (Fig. 4a,b) directly demonstrating Rap1 regulation of the RIAM–MIT complex. Conversely, expression of activated Rap1(G12V) induced a dramatic increase (Fig. 4a,b) in BiFC. In sharp contrast, activated H-Ras(G12V) suppressed the BiFC signal (Fig. 4a,b), suggesting that the previously described H-Ras inhibition of integrin activation^34^ is ascribable to disruption of an MIT complex. In sum, these data show that, the observed BiFC signal can report the physiological regulation of formation of the MIT complex in living cells.

### The MIT complex co-localizes with activated integrins

We stained non-permeabilized cells with an anti-α_IIb_β_3_ antibody to test whether the MIT complex was associated with cell surface integrins, resulting in staining of most of the BiFC-positive structures in the plasma membrane (Supplementary Fig. 2b,c). Furthermore, the BiFC-positive surface structures contained activated integrins, as they stained with PAC1, an activation-specific anti-α_IIb_β_3_ (ref. 35; Fig. 4c). However, the PAC1 staining was less apparent on the tips of filopodia suggesting that at this location some of the integrins were occupied by ligands that would displace the PAC1. Indeed, the BiFC-positive tips of filopodia were brightly stained with anti-LIBS1, an anti-α_IIb_β_3_ that recognizes the activated and ligand-bound integrin^36^,^37^ (Supplementary Fig. 5). As shown above (Fig. 1d; Supplementary Fig. 1d), BiFC reports only a subset of total integrins (Supplementary Fig. 5). Taken together, these data indicate that BiFC reports the presence of an MIT complex that co-localizes with cell surface-activated ligand-bound and unoccupied integrins.

### BiFC identifies diverse MIT complexes in multiple cell types

**Figure 5:**
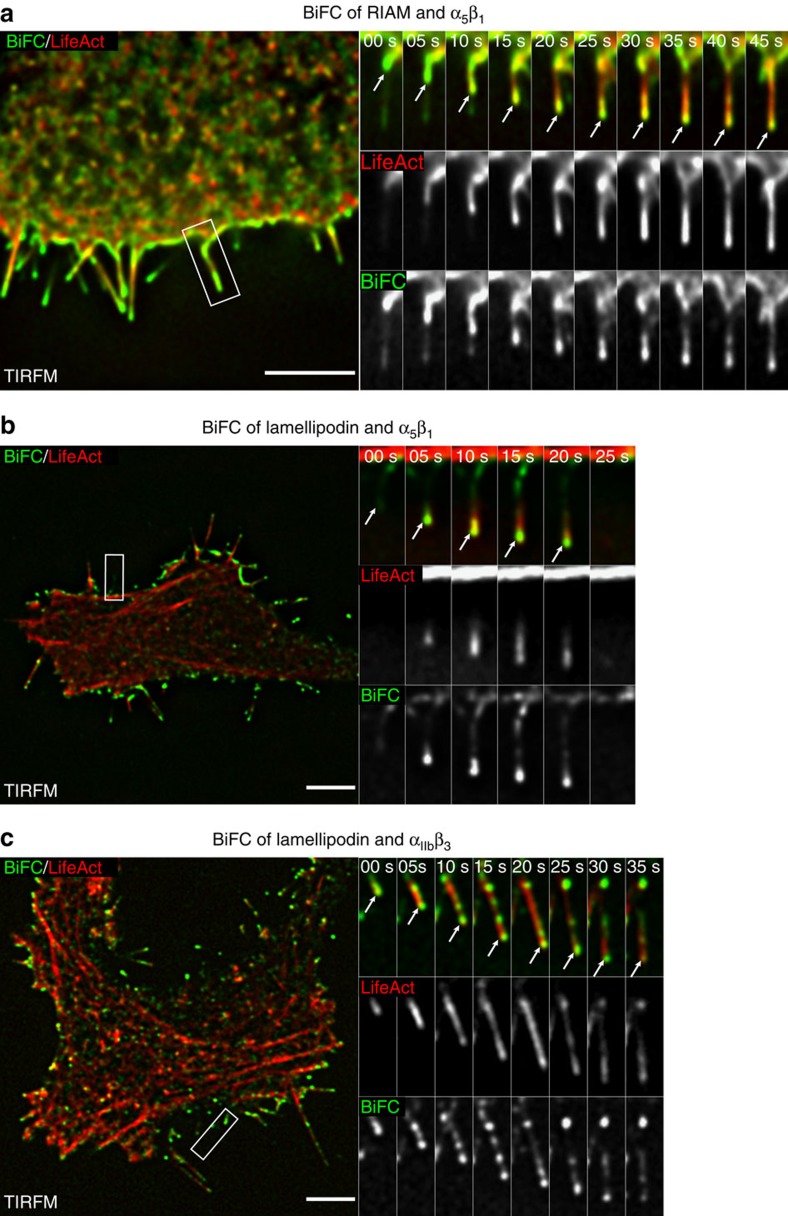
The MIT complex can form with multiple integrins and Lpd. (**a**) U2-OS cells expressing VN-RIAM, integrin α_5_-VCβ_1_ and mCherry-LifeAct were plated on fibronectin for 2 h. The cells were imaged with time-lapse TIRFM. A segment of the cell edge is enlarged and displayed as a movie montage at 5 s intervals. The arrows show the growth of an actin protrusion with BiFC enriched at its tip. Scale bar, 5 μm. (**b**) U2-OS cells expressing VN-Lpd, integrin α_5_-VCβ_1_, and mCherry-LifeAct were plated on fibronectin for 2 h. The cells were imaged with time-lapse TIRFM. A segment of the cell edge is enlarged and displayed as a movie montage at 5 s intervals. The arrows show the growth of an actin protrusion with BiFC enriched at its tip. Scale bar, 5 μm. (**c**) U2-OS cells expressing VN-Lpd, integrin α_IIb_-VCβ_3_, and mCherry-LifeAct were plated on fibrinogen for 2 h. The cells were imaged with time-lapse TIRFM. A segment of the cell edge is enlarged and displayed as a movie montage at 5 s intervals. The arrows show the growth of an actin protrusion with BiFC enriched at its tip. Scale bar, 5 μm.

Our initial analysis was conducted with the complex formed with RIAM and α_IIb_β_3_. We asked whether this approach could be used to visualize complexes of RIAM with other integrins, such as α_5_β_1_. In cells expressing α_5_-VC and VN-RIAM, we observed a similar localization of BiFC in protrusions (Fig. 5a; Supplementary Movie 8). In addition, we observed the α_IIb_β_3_/talin/RIAM complex in similar locales in a spectrum of cell lines (for example, PtK, HeLa, HT-1080 and NIH-3T3) and primary cells (for example, HuVEC, Mouse Embryonic Fibroblasts) (Supplementary Fig. 6a) and was also true with α_5_β_1_-RIAM (Supplementary Fig. 6b). Therefore, MIT complex formation and localization is similar in multiple cell and integrin types. We also found that the talin-binding RIAM paralogue, Lpd^19^,^24^,^38^, formed a complex with α_5_β_1_ (Fig. 5b; Supplementary Movie 9) and α_IIb_β_3_ (Fig. 5c; Supplementary Movie 10) at the tips of actin-based protrusions. In summary, either mammalian MRL protein can form a complex with talin and activated integrins localized at the tips of ‘sticky fingers’ and they can do so in multiple cell types.

### Assembly and localization of the MIT complex without BiFC

**Figure 6:**
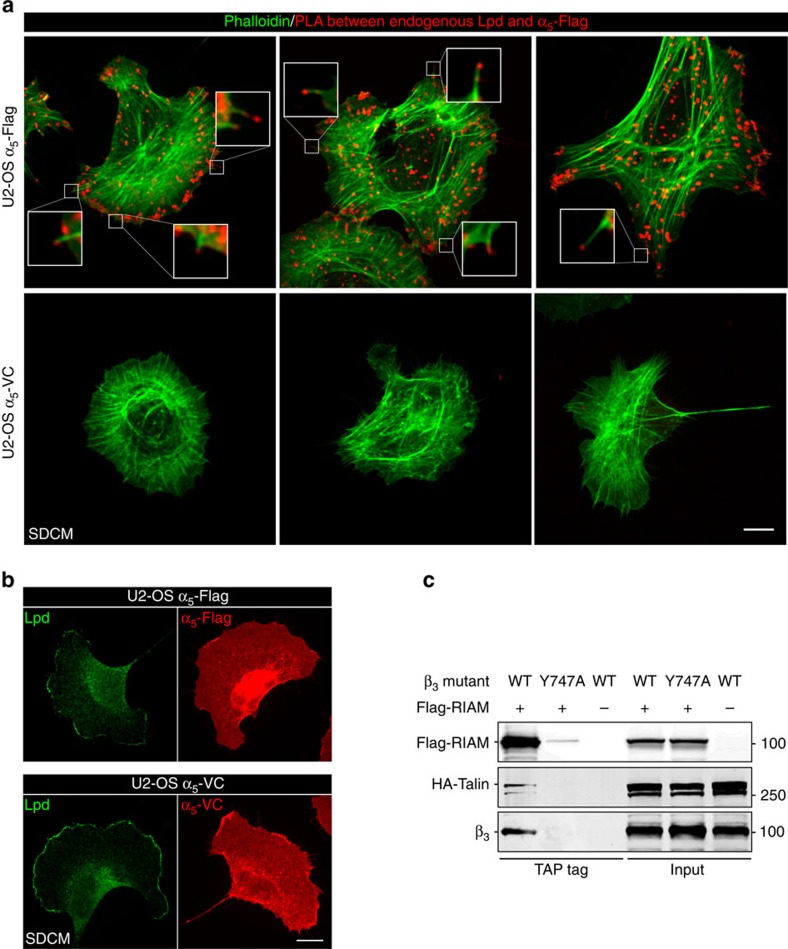
BiFC-independent approaches demonstrate assembly and localization of the MIT complex at the tips of sticky fingers. (**a**) U2-OS cells stably expressing either 3xFlag-tagged integrin α_5_ or VC-tagged integrin α_5_ were plated on fibronectin for 2 h, fixed, permeabilized, stained with both Flag and Lpd antibodies and proximity ligation assay was performed to assess co-localization of both endogenous Lpd and integrin α_5_. Cells were counterstained with phalloidin and imaged by spinning disk confocal microscopy (SDCM). The proximity ligation signal is enriched at the cell edge and present at the tip of filopodia-like protrusions. U2-OS cells expressing α_5_-VC were used as negative control to show the specificity of the signal. Three representative cells for each condition are shown. Scale bar, 10 μm. (**b**) Cells described in **a** were stained with the indicated antibodies and imaged by SDCM. Polyclonal anti-EGFP antibody was used to detect VC-tagged integrin α_5_. Note the distribution of endogenous Lpd is similar in cells expressing either α_5_-Flag or α_5_-VC. Scale bar, 10 μm. (**c**) Tandem affinity purification approach showing assembly of a RIAM–integrin–talin complex. U2-OS cells expressing 3xFlag-RIAM, HA-talin and either α_IIb_-SBPβ_3_ or α_IIb_-SBPβ_3_(Y747A) were harvested and homogenized. Flag-RIAM was purified with anti-Flag resin and bound proteins were eluted with Flag peptides prior to a second purification with streptavidin resin to isolate α_IIb_-SBP integrin. Isolated proteins were analysed by Western blot with anti-HA, anti-β_3_ and anti-Flag antibodies. The talin-binding defective integrin β_3_(Y747A) mutant blocked the formation of the RIAM-α_IIb_β_3_-talin complex.

The foregoing studies established that BiFC could be used to observe the localization, dynamics and regulation of assembly of the MIT complex in living cells. The interaction of VN and VC domains, required to form BiFC, can increase the stability of the observed complex^28^. We performed a proximity ligation assay to visualize the localization of the MIT complex in the absence of BIFC. To perform this assay, we used a rabbit anti-Lpd antibody to stain endogenous Lpd in U2-OS cells and stained Flag-tagged recombinant integrin α5 with a murine anti-Flag antibody (Fig. 6b). We observed localization of the proximity ligation signal to the ends of actin filaments in protruding areas at the cell edge and at the tips of filopodia (Fig. 6a). Consistent with previous studies reporting that this method only detects a subset of protein complexes^39^, only a fraction of filopodia exhibited a signal at their tips. We also used tandem affinity purification to biochemically assess the formation of a RIAM/integrin/talin complex in the absence of BiFC. Sequential affinity chromatography under non-denaturing conditions isolated Flag-tagged RIAM followed by streptavidin affinity chromatography isolated integrin α_IIb_-SBPβ_3_ in a complex containing talin. The formation of this complex required the talin–integrin interaction because none of these components were identified when the tandem affinity purification was performed from cells bearing talin-binding defective α_IIb_-SBPβ_3_(Y747A) mutant (Fig. 6c). Finally, when we examined the effect of BiFC on cellular behaviour, we observed BiFC-positive cells exhibited similar numbers and lengths of finger-like protrusions to cells lacking BiFC (Supplementary Fig. 7). In summary, the presence of BiFC, which signals the formation of the MIT complex did not alter protrusion formation and, in the absence of BiFC, the MIT forms is localized to the ends of actin filaments in cell protrusions.

### An MIT complex forms without ligand binding

**Figure 7:**
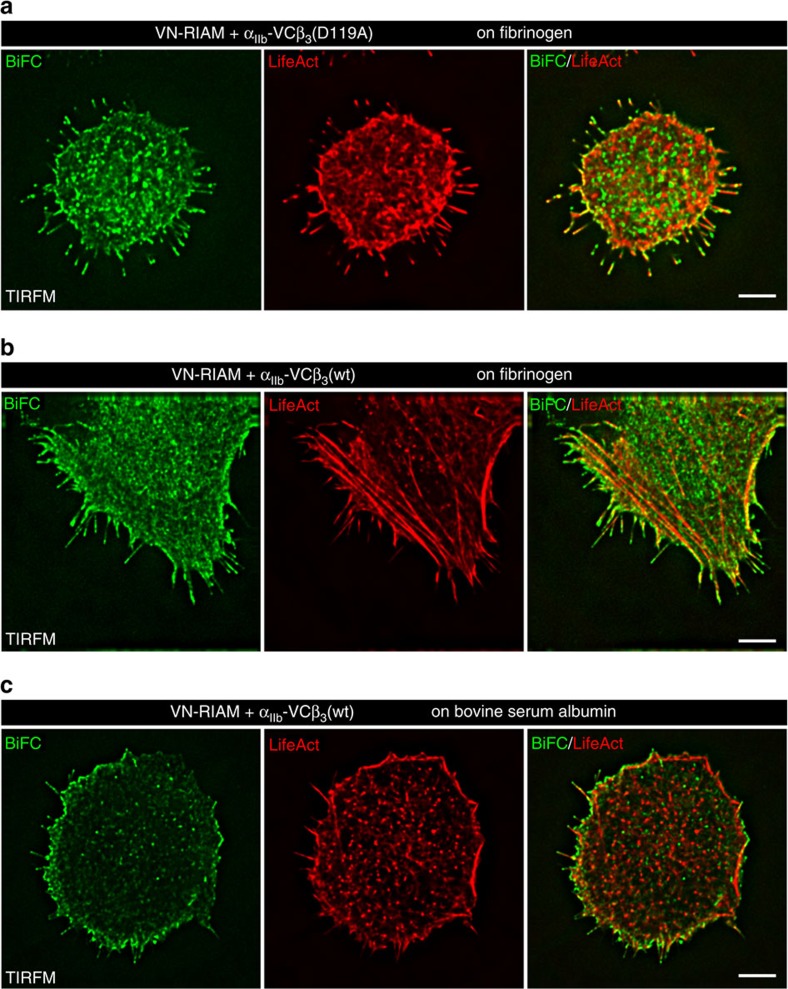
The MIT complex forms without ligand engagement. (**a**) U2-OS cells expressing VN-RIAM and ligand-binding defective integrin α_IIb_-VCβ_3_(D119A) were plated on fibrinogen for 2 h. The cells were imaged with TIRFM. Note lack of spreading documents lack of ligand engagement and because the cells are not spread, filopodia are at varying angles to the TIRF plane. Scale bar, 5 μm. (**b**,**c**) U2-OS cells expressing VN-RIAM and integrin α_IIb_-VCβ_3_ were plated on fibrinogen (**b**) or BSA (**c**) for 2 h. The cells were imaged with TIRFM. Cells appear well spread with discernible actin stress fibers on fibrinogen. Unspread cells on BSA still exhibit filopodia and BiFC. Scale bar, 5 μm.

One of the hallmarks of ‘sticky fingers’ is that they contain activated unoccupied integrins. As noted above, BiFC that reported formation of the α_IIb_β_3_ MIT complex co-localized with PAC1 (Fig. 4c), a ligand-mimetic antibody, suggesting that the MIT complex forms without ligand binding. To directly test this idea, we used β_3_(D119A), a ligand binding-defective mutant^40^. Even though cells expressing α_IIb_-VC β_3_(D119A) did not spread on fibrinogen, they still formed actin-containing microspikes that exhibited bright BiFC at their tips (Fig. 7a; Supplementary Movie 11). Alternatively, we examined cells expressing α_IIb_-VCβ_3_ plated on non-adhesive bovine serum albumin (BSA). Cells on fibrinogen spread well (Fig. 7b), but cells on BSA did not. Nevertheless, BiFC was still evident at the tips of actin-based protrusions in cells (Fig. 7c) plated on BSA. Thus, the MIT complex forms without ligand engagement to generate activated but unoccupied integrins at the tips of actin-based protrusions.

### RIAM in the MIT complex contributes to protrusion

**Figure 8:**
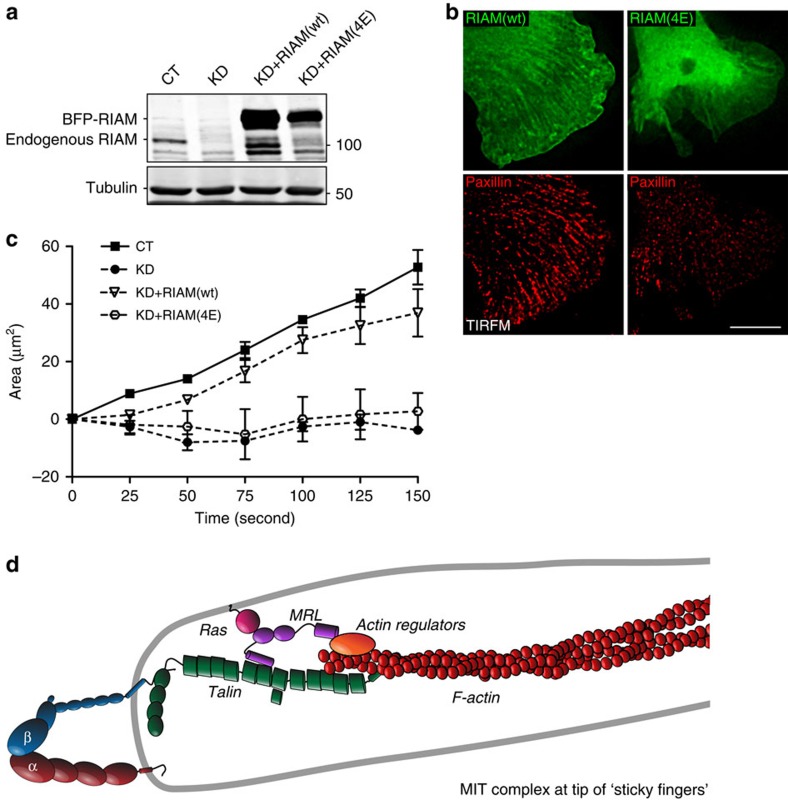
The MIT complex directs protrusion. NIH-3T3 cells expressing the membrane marker mCherry-K-Ras-Caax and either control shRNA (CT) or RIAM shRNA (KD) were transiently transfected with constructs encoding BFP-RIAM(WT), talin-binding defective BFP-RIAM(4E) mutant or BFP alone. (**a**) Cell lysates were analysed by Western blot with anti-RIAM antibody. Tubulin was used as a loading control. (**b**) Cells were plated on fibrinogen for 2 h, fixed, stained with anti-paxillin antibody and imaged with TIRFM. Scale bar, 10 μm. (**c**) Cells were plated on fibrinogen for 2 h and imaged at 5 s interval for 3 min. An actively protruding part of the cell edge was computationally detected as described in Methods, and the changes in protrusion area over time were plotted. Each point depicts the mean ± s.e.m. of three independent experiments. A total of 3–5 cells for each condition have been analysed in each experiment. Two-way analysis of variance with Bonferroni’s test were used to compare the populations: KD versus CT, *P*<0.001; KD+RIAM(WT) versus CT, P=NS; KD+RIAM(4E) versus CT, *P*<0.001. (d) The MIT complex is the molecular basis of ‘sticky fingers’ at the leading edge. The N terminus of the MRL protein binds and recruits talin to the integrin to induce activation. The C terminus of MRL protein increases processive actin polymerization in part by recruiting ENA/VASP, thereby propelling the movement of the ‘sticky fingers’.

As noted above, MRL proteins drive cell protrusion and play a role in cell migration and we have shown here that they form an MIT complex at the tips of protrusive structures that help guide migration. Because MRL proteins may have multiple functions, we asked if their capacity to enter the MIT complex enabled them to support cell protrusion. To test this idea, we silenced RIAM in NIH-3T3 cells and rescued its expression with a mutant in the talin-binding RIAM(4E) N-terminal domain that preserves binding sites for Rap1 and proline-rich sequences that interact with VASP^24^ (Fig. 8a). Blocking the RIAM–talin interaction led to a dramatic reduction in RIAM recruitment to paxillin-staining adhesions (Fig. 8b). As previously reported^23^, silencing RIAM markedly inhibited lamellipodial protrusion; rescue with wild-type RIAM restored protrusion whereas the RIAM(4E) mutant had no effect (Fig. 8c; Supplementary Movie 12). Therefore, RIAM in the MIT complex supports cellular protrusion.

## Discussion

Rapidly translocating activated integrins associated with growing actin filaments are proposed to guide cell migration and have been termed^10^ ‘sticky fingers’ at the leading edge. Here we have characterized the molecular basis of these sticky fingers using an indirect BiFC approach to visualize an MIT complex of MRL proteins with talin and activated integrins in lamellipodia and filopodia. The MIT complex is enriched at the tip of growing actin filaments in protrusions. Formation of this complex required the capacities of integrins to bind talin and the complex was regulated by the activity of a Ras family GTPase. Moreover, disruption of the MIT complex using a RIAM mutant defective in talin binding led to a severe impairment in cell protrusion. In summary, these data reveal the molecular basis of formation of ‘sticky fingers’ at the leading edge and show that the MIT complex is important in forming these protrusions that guide cell migration.

Here we modified BiFC, a well-established method to study direct protein–protein interactions^41^, to identify the ternary MIT complex and to study its localization and regulation. We validated the indirect nature of BiFC between the MRL protein and the integrin by showing that it was critically dependent on the capacity of each of them to bind to a talin bridge. The desire to image a multi-molecular complex led us to employ BiFC rather than Förster Resonance Energy Transfer, whose signal decays exponentially above a distance of 10 nm between the interacting proteins^42^, because the talin head domain itself has a diameter exceeding 10 nm (ref. 43). In contrast, BiFC can work over longer distances and is less reliant on the orientation of the interactors. Although the interaction of VN and VC domains can prolong complex stability^28^, the talin-dependence of BiFC reported here establishes its specificity. Moreover, we ensured that the BiFC itself does not enforce the formation and the localization of the MIT complex. First, we used a split tandem affinity approach to biochemically demonstrate a RIAM/integrin/talin complex without the BiFC moieties. Second, we used a proximity ligation assay in fixed cells to demonstrate the formation of the MIT complex between α_5_β_1_ integrin and endogenous lamellipodin, and showed that it is enriched at the cell edge and the tips of actin bundles in protrusions. In addition, since BiFC required expression of recombinant proteins, we took care to analyse cells whose morphology and morphodynamics were not divergent from control untransfected cells to exclude overexpression artifacts as before^44^. Thus indirect BiFC can be used to visualize higher order protein–protein interactions and we have here established that it provides a means to identify the MIT complex in living cells.

The MIT complex was largely co-localized with a subset of cell surface-activated integrins, as most of the integrins were accessible to antibodies in intact cells. Importantly, the fact that some of the BiFC-associated integrins bound PAC1 shows that they were activated and unoccupied, since PAC1 is a true ligand-mimetic antibody, that is, it will not bind to a ligand-occupied integrin^35^. Furthermore, the MIT complex formed with ligand binding-defective β_3_(D119A), substantiating that it can form with unoccupied integrins and suggesting that the MIT complex is the molecular identity of the ‘adhesion precursor complex’ proposed by Choi *et al*.^45^ and forms the talin in nodes at the edge of the lamellipodium described by DePasquale and Izzard^46^. Similarly, ligand binding does not disassemble the MIT complex, since BiFC co-localized with an occupancy-dependent antibody, anti-LIBS1 (ref. 36), in filopodial tips. Importantly, we observed bound PAC1 not associated with BiFC, suggesting that either these activated integrins were generated by endogenous MRL proteins or by MRL protein-independent mechanisms of activation, for example, vinculin^23^,^31^. Furthermore, the absence of detectable vinculin from the tips of protrusions highlights the value of selective visualization of integrins activated by a specific mechanism. Previous methods for detecting activated integrins utilized either binding of natural ligands or their fragments or conformation-specific antibodies^47^ and could not differentiate between different activation pathways. Since BiFC enables visualization of only a subset of integrins activated by a specific mechanism, it can form the basis of convenient screens for modulators of specific integrin activation pathways. Finally, we note the surprising absence of substantial vesicular BiFC, in spite of the extensive integrin trafficking to and from the plasma membrane^48^. Because BiFC maturation is time dependent^49^ we cannot exclude the possibility of intracellular sites of assembly of the MIT complex. In sum, the detected MIT complex is largely at the plasma membrane, can contain both occupied and unoccupied activated integrins, and represents a molecularly defined ‘modular’ subset of such integrins.

Association of activated translocating integrins with the tips of growing actin filaments are a hallmark of ‘sticky fingers’^10^ and is a striking feature of MIT complex as visualized here. The ‘sticky fingers’ appear to be propelled by actin polymerization and like listeria, MRL proteins promote actin polymerization in part through recruitment of ENA-VASP proteins and activators of the Arp2/3 complex to proline-rich motifs^17^,^21^,^25^. Thus, the MRL protein N terminus can recruit and stabilize talin at the membrane in a Ras GTPase-dependent manner to activate integrins^22^ and the MRL protein C terminus that can promote actin polymerization^17^,^21^,^25^ to drive the rapid translocation of the integrin (Fig. 8d). Together, these two biochemical functions of the MRL proteins result in the formation of the ‘sticky fingers’ at the leading edge that direct protrusion during cell migration.

## Methods

### Antibodies and reagents

Rabbit anti-Flag (F7425), mouse anti-talin (8d4; T3287), mouse anti-vinculin (hVIN-1; V9131) and mouse anti-tubulin (DM1A; T9026) antibodies were purchased from Sigma-Aldrich. Mouse anti-HA (16B12; MMS-101R) was purchased from Covance. Polyclonal rabbit anti-full-length EGFP antibody (632592) was from Clontech. Mouse monoclonal antibodies PAC1 (ref. 35), anti-LIBS1 (ref. 36) and anti-α_IIb_β_3_ (D57)^36^, and rabbit polyclonal anti-β_3_ (Rb8053)^50^ were described before. Rabbit anti-RIAM antibody was generously provided by V. Boussiotis (Dana-Farber Cancer Institute, Boston, MA, USA). Rabbit anti-Lpd antibody was a generous gift from F. Gertler (Massachusetts Institute of Technology, Cambridge, MA, USA). Rabbit anti-GAPDH (FL-335; sc-25778) was from Santa Cruz Biotechnology. Goat secondary antibodies conjugated to Alexa Fluor 568 (A-11031; A-11036) and 647 (A-21236; A-21245) were purchased from Molecular Probes (Life Technologies).

### Plasmids

BiFC Venus vector encoding for α_IIb_-VC was described previously^38^. The sequence encoding the streptavidin binding peptide (SBP) was amplified by PCR and then inserted in-frame into the 3′ end of α_IIb_ after removing VC in pcDNA3.1-α_IIb_-VC to form α_IIb_-SBP. To generate the α_5_-VC construct, a plasmid template encoding human integrin α_5_ was amplified by PCR to place appropriate restriction sites, and the PCR product was inserted in-frame at the 5′ end of VC in pcDNA3.1 (Life Technologies). The N-terminal half of Venus was amplified to place appropriate restriction sites, and the PCR product was inserted in-frame into the 5′ end of RIAM after removing EGFP in pEGFP(C1)-RIAM to form VN-RIAM. The sequence encoding for the 3xFlag tag (DYKDHDGDYKDHDIDYKDDDDK ) was amplified to place appropriate restriction sites, and the PCR product was inserted in-frame into the 3′ end of RIAM to generate the VN-RIAM-Flag construct. To generate the VN-Lpd construct, the sequence encoding for human Lpd was amplified to place appropriate restriction sites, and the PCR product was inserted in-frame into the 3′ end of VN after removing RIAM in the vector encoding for VN-RIAM. VN-RIAM was subcloned into the pTRE2-hyg expression vector (Clontech) by standard techniques. Integrin β_1_, β_3_, β_3_(L746A), β_3_(Y747A), β_3_(D119A) and β_3_(D119A, Y747A) were constructed in pcDNA3.1 by PCR-based cloning and site-directed mutagenesis. cDNAs encoding β_3_, β_3_(L746A) and β_3_(Y747A) were then amplified by PCR and subcloned into PRRL-CMV^44^. RIAM, Rap1A(V12), Rap1GAP1, H-Ras(V12) and ubiquitin were amplified by PCR and subcloned into p3xFLAG-CMV-7.1 (Sigma-Aldrich). K-Ras-CAAX was amplified by PCR and ligated into both pTagBFP-N (Evrogen, Moscow, Russia) and PRRL-CMV-mCherry. A cDNA encoding shRNA-resistant RIAM was generated by PCR and cloned into pTagBFP-N. Talin-binding defective RIAM(4E)^24^ mutant (M11E, F12E, L15E and L16E) was generated by PCR-based cloning and *in situ* mutagenesis using BFP-RIAM(WT)^23^ as template. The mutations were verified by DNA sequencing. Plasmids encoding shRNA for mouse RIAM and the human U6 promoter^25^ were amplified by PCR to place Xba1 and Xho1 sites, and PCR products were cloned into lentiviral vector FG12. Promoter and shRNA sequences were confirmed by DNA sequencing. Control shRNA was obtained from a scrambled sequence of mRNA derived from Rock1 (ref. 11). RIAM, vinculin and talin were fused to mCherry and inserted into pcDNA3.1 by PCR-based cloning. Human full-length talin was fused to a HA-tag at the N terminus^51^. Integrin β_3_ was amplified by PCR and then inserted at the 5′ end of mCherry into pcDNA3.1. mCherry-LifeAct was a gift from O Pertz (University of Basel, Switzerland). EGFP-Myosin-X construct was a gift from R Cheney, University of North Carolina. Myosin-X cDNA was fused to mCherry and inserted into pcDNA3.1 by PCR-based cloning.

### Cell lines, transfection and transduction

U2-OS, HT-1080, NIH-3T3 and MEF cells were purchased from ATCC. Cells were cultured in DMEM (Mediatech) supplemented with 10% fetal bovine serum (FBS), nonessential amino acids, L-glutamine, 100 U ml^−1^ penicillin and 100 μg ml^−1^ streptomycin. Tet-On U2-OS cells were grown in DMEM supplemented with 10% Tet-Free FBS (Clontech), 100 U ml^−1^ penicillin, 100 μg ml^−1^ streptomycin, 100 μg ml^−1^ G418 (Life Technologies) and 100 μg ml^−1^ Hygromycin B (Life Technologies). Female rat kangaroo kidney epithelial (PtK1) cells were a gift from C. Waterman (The National Heart, Lung, and Blood Institute, USA) and cultured in F-12 medium (Life Technologies) supplemented with 10% FBS. HuVEC were purchased from Lonza and cultured in EGM-2 medium (Lonza). All cells were maintained under standard humidified incubator conditions at 37 °C and 5% CO_2_. Cell lines were routinely checked for Mycoplasma by PCR.

U2-OS and NIH-3T3 cells were transiently transfected with FuGene HD (Promega) or TransIT-LT1 (Mirus) according to the manufacturer’s protocol. Transient transfection of MEF, PtK1 and HuVEC were carried out using Amaxa nucleoporator (Lonza). Cells were washed in PBS and resuspended in 100 μl of PBS containing 3–5 μg of plasmid constructs and electroporated using programs A034 (HuVEC), A023 (MEF) or T020 (PtK). The cells were allowed to spread for 1–2 h before they were washed with fresh medium and allowed to grow overnight.

FG12 lentiviral constructs encoding RIAM shRNA and control have been previously described^22^. VSV-G pseudotyped lentiviral vectors were produced by transient transfection of HEK293T (human embryonic kidney) cells. Viral particles from cell culture supernatant were used for infection of U2-OS or NIH-3T3 cells overnight. The cells were assayed at least 4 days after transduction.

To generate U2-OS inducible cell lines, a total of 1 × 10^6^ U2-OS Tet-On cells (Clontech) expressing the tetracycline-controlled transactivator were plated into a 100 mm dish and transfected with pTRE2-hyg-VN-RIAM using TransIT-LT1 (Mirus). Forty-eight hours after transfection, 500 μg ml^−1^ of Hygromycin B (Life Technologies) was added. Transformants were selected and single cells cloned using the limiting dilution method. To test for induced expression, 200 ng ml^−1^ doxycycline (Sigma-Aldrich) was added to the medium and VN-RIAM-positive clones were selected by flow cytometry analysis using a polyclonal anti-EGFP antibody (cross-reacts with VN). Selected clones were transduced with lentiviral particles encoding α_IIb_-VCβ_3_(WT), α_IIb_-VCβ_3_(L746A) or α_IIb_-VCβ_3_(Y747A) and assayed as a polyclonal population. BiFC-positive cells expressing α_IIb_-VCβ_3_(WT) were single cell cloned by automated cell deposition unit using a BD FACSAria after induction of VN-RIAM expression with 200 ng ml^−1^ doxycycline overnight.

### Tandem affinity purification

Integrins α_IIb_-SBP, β_3_(D119A), 3xFlag-RIAM and HA-talin were transiently transfected into U2-OS cells. To assess the importance of talin binding to the integrin, β_3_(D119A,Y747A) was substituted for β_3_(D119A). Twenty-four hours later, cells were homogenized in lysis buffer (50 mM Tris-HCl pH7.4, 100 mM NaCl, 0.5% NP-40, 10 mM N-Ethylmaleimide, 0.5 mM MgCl_2_, 0.5 mM CaCl_2_, 1 μM calpeptin, protease inhibitors cocktail) and incubated with anti-Flag antibody resin (Sigma-Aldrich) for 4 h at 4 °C and eluted with 200 μg ml^−1^ Flag peptide. The elute was added to streptavidin resin (streptavidin plus ultralink resin, Thermoscientific) and incubated for 2 h at 4 °C. Bound proteins were washed and boiled in Laemmli buffer for SDS–polyacrylamide gel electrophoresis and Western blot analysis. All antibodies used for Western blot were diluted 1:1,000. Uncropped scans of Western blots are displayed in Supplementary Fig. 8.

### Flow cytometry

Cells were harvested by trypsinization, washed with PBS and then fixed in 2% paraformaldehyde for 10 min. After washings, cells were transferred into permeabilization buffer (PBS supplemented with 0.5% BSA and 0.5% (wt:vol) saponin) for 20 min. Cells were centrifuged, resuspended in permeabilization buffer containing primary antibodies (anti-Flag or D57 antibodies; 1:200) on ice for 30 min, washed three times in permeabilization buffer, and incubated in permeabilization buffer containing goat anti-mouse antibody coupled to Alexa Fluor 647 (1:300) for 30 min on ice, washed in permeabilization buffer, and then resuspended in PBS to get analysed on a BD FACSCalibur (BD Biosciences, San Diego, CA, USA) using CellQuest software (BD Biosciences). Results were expressed as the average mean fluorescence intensity corresponding to BiFC within the Flag- or D57-positive cell population.

### Immunofluorescence microscopy

Glass coverslips were coated with 10 μg ml^−1^ fibronectin or fibrinogen at room temperature for 1 h. Cells were adhered to the indicated substrate for the indicated period of time at 37 °C. The cells were fixed with 2% formaldehyde, in 100 mM PIPES, 1 mM EGTA, 1 mM MgSO_4_ for 30 min at 37 °C. When permeabilization is required, cells were incubated with 0.2% TritonX-100 in PBS for 10 min. Following blocking with 10% goat serum in PBS for 10 min, cover slips were incubated with the primary antibody (1:200) for 2–3 h. The cells were washed three times with PBS, incubated with the secondary antibody for 1 h, washed and mounted with Fluorsave mounting reagent (Calbiochem, La Jolla, CA, USA). For PAC1 staining, cells were incubated with serum-free medium for 20 min and PAC1 antibody was added (1:100 dilution) for 10 min before fixing the cells with 4% paraformaldehyde in PBS for 10 min. The cells were then incubated with the secondary antibody (1:300) for 20 min before being washed with PBS and mounted onto a glass slide with Fluorsave mounting reagent.

The slides were imaged with an IX81 inverted microscope (Olympus, Tokyo, Japan) equipped with a CSU-X1-A1 spinning-disk head (Yokogawa, Tokyo, Japan), electron-multiplying CCD 14 bit 1K × 1K camera (Hamamatsu, Hamamatsu, Japan), four laser lines (405, 488, 560 and 640 nm), and software controlled by Volocity (PerkinElmer, Waltham, MA, USA). When multiple conditions were imaged as part of the same experiment, the laser power, camera sensitivity and camera exposure times were kept constant. Post-acquisition processing was limited to adjusting contrast or correcting registration by Volocity (PerkinElmer, Waltham, MA, USA). Any modifications were kept consistent when multiple conditions were processed as part of the same experiment.

Line scans were performed with ImageJ (NIH). Briefly, a line was drawn along a filopodium from its tip to a few μm beyond the cell membrane. The intensity values were extracted from ImageJ and input into Microsoft Excel to plot as a multi-colour curve.

### Spinning disc confocal microscopy

For live cell imaging, the cells were plated on fibrinogen or fibronectin-coated glass cover slips for the desired time. The cover slips were transferred to a Chamlide magnetic chamber (CMB for 25 mm round coverslips; Live cell instruments, Seoul, Korea) and immersed in culture medium. The chamber was incubated with 5% CO_2_ at 37 °C for live imaging. The cells were imaged with the IX81 inverted microscope as described for immunofluorescence microscopy. Typically the cells were imaged with the × 60 objective (numerical aperture (NA) 1.42) for 1–3 min at 5 s intervals. Post-acquisition processing was limited to adjusting contrast, image cropping or correcting registration by Volocity (PerkinElmer, Waltham, MA, USA). Modifications were applied equally to all conditions in a given experiment.

### Total internal reflection fluorescence microscopy

Cells were plated on cover slips as described above and incubated at 37 °C in 5% CO_2_ and visualized with the Applied Precision OMX DeltaVision Ring TIRF module (Issaquah, WA, USA) using a × 100 objective (NA 1.40) with four laser lines (405, 488, 562 and 641 nm). The cells were imaged for 1–3 min at 5 s intervals. After acquisition, the data sets were transferred to a Linux workstation employing SoftWoRx software (Applied Precision) that uses an algorithm experimentally produced on the system from the convolution of a point spread function. All data sets were subjected to eight deconvolution iterations to reduce extraneous light or scattered light captured by the camera. Subsequent image processing and analysis was done with Volocity.

### Fluorescence fluctuation imaging and cross-variance analysis

Cross-variance analysis was performed on TIRF image time series acquired using an Olympus IX71 microscope equipped with a 100 × 1.45 NA Plan Apo total internal reflection fluorescence microscopy (TIRFM) oil objective, a Ludl controller (Ludl Electronic Products, Hawthorne, NY, USA), and Metamorph Software (Molecular Devices, Downingtown, PA, USA). mGFP and mCherry excitation was produced using the 488 nm and 568 nm lines of an Ar-Kr ion laser (Melles Griot, Albuguergue, NM, USA), respectively. A polychroic mirror (Z488/568 rpc), dual emission filter (Z488/568 nm; Chroma Technology, Bellows Falls, VT, USA), and a Dual-View (Photometrics, Tucson, AZ, USA) were used for simultaneous dual-colour image acquisition. Images were collected on a QuantEM: 512C EMCCD camera (Photometrics) in non-overlapping mode at 3X conversion gain and 700–1,000 EM gain, Image exposure time and delay time between images were 100 and 500 ms, respectively.

To detect molecular interaction using fluorescence fluctuation cross-variance analysis, we calculate the cross-variance (*B*_cc_) parameter at a given pixel location 
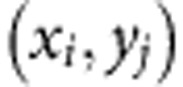
 for given time segments as:




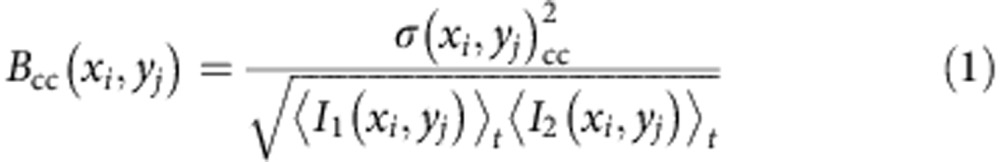





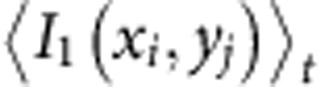
 and 
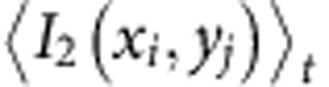
 are the fluorescence average intensity for channels 1 and 2. 
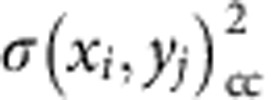
 is the fluorescence intensity cross-variance between channels 1 and 2 for time, *t*, and is defined as:




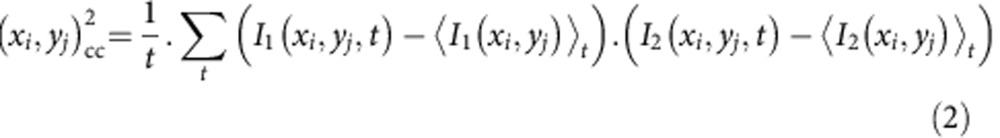





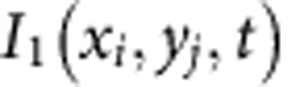
 and 
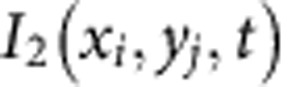
 are the fluorescence pixel intensity at time, 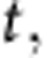 for channels 1 and 2, respectively.

If molecules represented by fluorescence intensity in two channels (wavelengths), channel 1 and 2, are non-interacting, their fluorescence fluctuations will be independent and the cross-variance will be centred around zero. In contrast, interacting species will produce positive (*B*_cc_) values. As the negative control, GAP-mGFP and GAP-mCherry, which localize independently to the cell membrane, are not known to interact and therefore do not display positive cross-variance values. As a positive control, mCherry-paxillin-mGFP; an adhesion marker tagged with two fluorescent probes and therefore produces correlated movement and positive cross-variance^33^.

### Duolink *in situ* proximity ligation assay

U2-OS cells expressing either α_5_-3xFlag or α_5_-VC were plated on fibronectin for 2 h, fixed in 4% paraformaldehyde, permeabilized with 0.2% Triton X-100 and stained with rabbit anti-Lpd and mouse anti-Flag antibodies (1:200). Hybridization, ligation, amplification and detection were realized according to the manufacturer’s instructions (Olink Bioscience). The cells were imaged with the IX81 inverted microscope with the × 60 objective (NA 1.42) as described for Immunofluorescence Microscopy. Post-acquisition processing was limited to adjusting contrast, image cropping or correcting registration by Volocity (PerkinElmer, Waltham, MA, USA). Modifications were applied equally to all conditions in a given experiment.

### Cell edge tracking

NIH-3T3 cells expressing either control shRNA or RIAM shRNA, each marked by EGFP expression, were transduced with lentiviral particles encoding mCherry-K-Ras-Caax and transiently transfected with vectors encoding BFP, BFP-RIAM(WT) or BFP-RIAM(4E). Cells were plated on fibrinogen-coated glass coverslips for 2 h and imaged with the IX81 inverted microscope, as described for live cell imaging, by an investigator blinded to the conditions. Typically the cells were imaged with the × 60 objective (NA 1.42) for 3 min at 5 s intervals. Detection analysis for the cell edge at each time point was performed as described previously^23^, using a custom-built software package written in Matlab^52^.

### Statistical analyses

Statistical analyses were performed using GraphPad Prism software. Statistical significance was determined by ANOVA with Bonferroni’s *post hoc* test, Mann–Whitney Rank sum test, or Student’s *t*-test. The test used for each experiment is indicated in the corresponding figure legend. All experiments have been reproduced at least three times.

## Additional information

**How to cite this article:** Lagarrigue, F. *et al*. A RIAM/lamellipodin–talin–integrin complex forms the tip of sticky fingers that guide cell migration. *Nat. Commun.* 6:8492 doi: 10.1038/ncomms9492 (2015).

## Supplementary Material

Supplementary Information Supplementary Figures 1-8

Supplementary Movie 1 The MIT complex localizes to the tips of finger-like protrusions. U2-OS cells expressing VN-RIAM, integrin αIIb-VCβ3 and mCherry-LifeAct were plated on fibrinogen for 2 h before imaging BiFC with time-lapse TIRFM at 5 sec intervals. One protrusion is magnified and displayed on the right. Scale bar: 5 μm.

Supplementary Movie 2 The MIT complex localizes to the tip of filopodia. U2-OS cells expressing VN-RIAM, integrin αIIb-VCβ3 and mCherry-LifeAct were plated on fibrinogen for 2 h before imaging BiFC with time-lapse spinning disk confocal microscopy (SDCM) at 5 sec intervals. Scale bar: 10 μm.

Supplementary Movie 3 The MIT complex localizes to lamellipodia and microspikes in NIH-3T3 cells. NIH-3T3 cells expressing VN-RIAM, integrin αIIb-VCβ3 and mCherry-LifeAct were plated on fibrinogen for 2 h before imaging BiFC with time-lapse spinning disk confocal microscopy (SDCM). Note the MIT complex is enriched at the edge of the lamellipodium in association with growing actin filaments. Scale bar: 5 μm.

Supplementary Movie 4 The MIT complex localizes to lamellipodia and microspikes in PtK1 cells. PtK1 cells expressing VN-RIAM, integrin αIIb-VCβ3 and mCherry-LifeAct were plated on fibrinogen for 2 h before imaging BiFC with time-lapse spinning disk confocal microscopy (SDCM). Scale bar: 10 μm.

Supplementary Movie 5 The MIT complex localizes to lamellipodia and microspikes in PtK1 cells. PtK1 cells expressing VN-RIAM, integrin αIIb-VCβ3 and mCherry-LifeAct were plated on fibrinogen for 2 h before imaging BiFC with time-lapse TIRFM. Note the striking localization of BiFC at the tips of actin filaments traversing the lamellipodia. Scale bar: 5 μm.

Supplementary Movie 6 BiFC co localizes with talin in at the tips of protrusions. U2-OS cells expressing VN-RIAM, integrin αIIb-VCβ3 and mCherry-talin were plated on fibrinogen for 2 h before imaging BiFC with time-lapse TIRFM. One finger-like protrusion is magnified and displayed on the right. Scale bar: 5 μm.

Supplementary Movie 7 The MIT complex does not co localize with vinculin in protrusion tips. U2-OS cells expressing VN-RIAM, integrin αIIb-VCβ3 and mCherry-vinculin were plated on fibrinogen for 2 h before imaging BiFC with time-lapse TIRFM. One protrusion is magnified and displayed on the right. Scale bar: 5 μm.

Supplementary Movie 8 The MIT complex forms with α5β1 and localizes to tips of protrusions. U2-OS cells expressing VN-RIAM, integrin α5-VCβ1 and mCherry-LifeAct were plated on fibronectin for 2 h before imaging BiFC with time-lapse TIRFM. One protrusion is magnified and displayed on the right. Scale bar, 5 μm.

Supplementary Movie 9 The MIT complex forms with α5β1 and Lpd to protrusion tips. U2-OS cells expressing VN-Lpd, integrin α5-VCβ1 and mCherry-LifeAct were plated on fibronectin for 2 h before imaging BiFC with time-lapse TIRFM. One protrusion is magnified and displayed on the right. Scale bar: 5 μm.

Supplementary Movie 10 The MIT complex forms with αIIb-VCβ3 and Lpd in protrusion tips. U2-OS cells expressing VN-Lpd, integrin αIIb-VCβ3 and mCherry-LifeAct were plated on fibrinogen for 2 h before imaging BiFC with time-lapse TIRFM. One protrusion is magnified and displayed on the right. Scale bar: 5 μm.

Supplementary Movie 11 The MIT complex forms without ligand engagement. U2-OS cells expressing VN-RIAM, integrin αIIb-VCβ3(D119A) and mCherry-LifeAct were plated on fibrinogen for 2 h before imaging BiFC with time-lapse TIRFM at 5 sec intervals. Two representative cells are shown. Scale bar: 5 μm.

Supplementary Movie 12 The MIT complex drives lamellipodial protrusion. NIH-3T3 cells expressing the membrane marker mCherry-K-Ras-Caax and either control shRNA (CT) or RIAM shRNA (KD) were transiently transfected with constructs encoding BFP-RIAM(WT), talin-binding defective BFP-RIAM(4E) mutant or BFP alone. Cells were plated on fibrinogen for 2 h and imaged by spinning disk confocal microscopy (SDCM) at 5 sec interval for 3 min. Scale bar: 5 μm.
