# Supplementary material for: A RIAM/lamellipodin–talin–integrin complex forms the tip of sticky fingers that guide cell migration
Source: Nat Commun. 2015 Sep 30;6:8492. doi: 10.1038/ncomms9492 (PMC4589889; doi:10.1038/ncomms9492)
Supplement: Supplementary Information — Supplementary Figures 1-8 [file ncomms9492-s1.pdf]

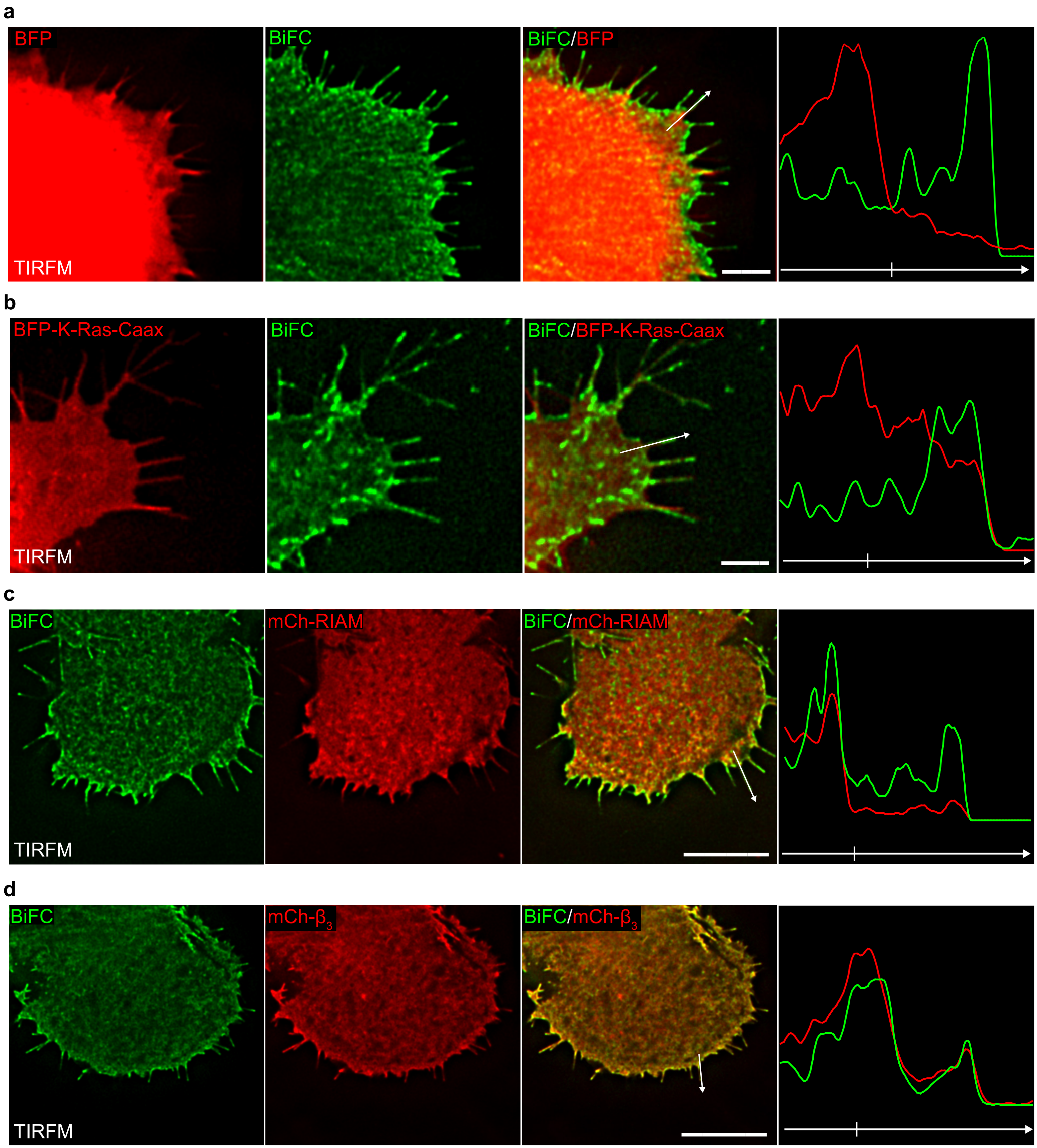

Supplementary Figure 1. BiFC marks a subset of both integrin and RIAM. (a,b) U2-OS cells expressing VN-RIAM and α_{IIb}-VCβ_3 and either the cytoplasmic marker blue fluorescent protein (BFP) (a) or the BFP joined to a plasma-membrane targeting motif (K-Ras-CAXX) (b). The cells were plated on fibrinogen for 2 h and then imaged with TIRFM. ImageJ software was used to perform a line scan from the base of the filopodium to its tip. Note the specific enrichment of BiFC signal at the tips of filopodia. Scale bar: 5 μm. (c,d) U2-OS cells expressing VN-RIAM, integrin α_{IIb}-VCβ_3 and either (c) mCherry-RIAM or (d) mCherry-β_3 integrin were plated on fibrinogen for 2 h before imaging with TIRFM. The line scans drawn along the filopodia exhibit the colocalization of BiFC with both mCherry-RIAM and mCherry-β_3. Note BiFC marks a subset of RIAM and integrin β_3 when imaging only the ventral surface of the cell by TIRFM. Scale bar: 10 μm.

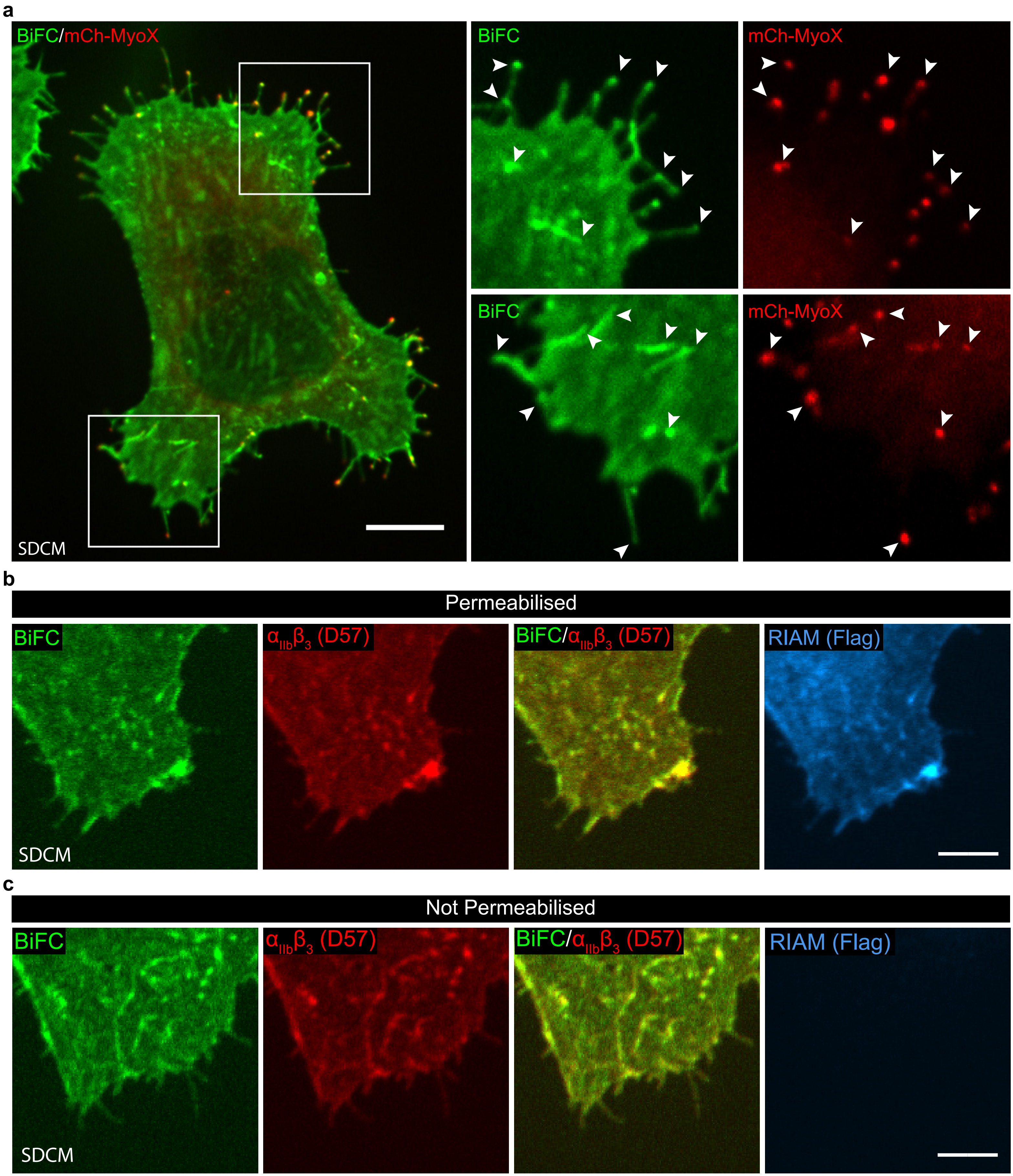

Supplementary Figure 2. The BiFC is enriched at the tips of sticky fingers. (a) U2-OS cells expressing VN-RIAM and α_{IIb}-VCβ_3 and mCherry-Myosin-X were plated on fibrinogen for 2 h, fixed and imaged by spinning disk confocal microscopy (SDCM). Note the colocalization of BiFC with Mysoin-X at the tips of filopodia extending from the cell edge or at the ventral surface facing down the substratum (arrowheads). Scale bar: 10 μm. (b,c) The MIT complex forms predominantly on the cell surface. U2-OS cells expressing VN-RIAM-Flag and integrin α_{IIb}-VCβ_3 were plated on fibrinogen for 2 h. The cells were fixed and either (b) permeabilized or (c) left unpermeabilized before staining for Flag and $\alpha_{IIb}\beta_3$ (D57). The cells were then imaged by spinning disk confocal microscopy (SDCM). Scale bar: 5 μm.

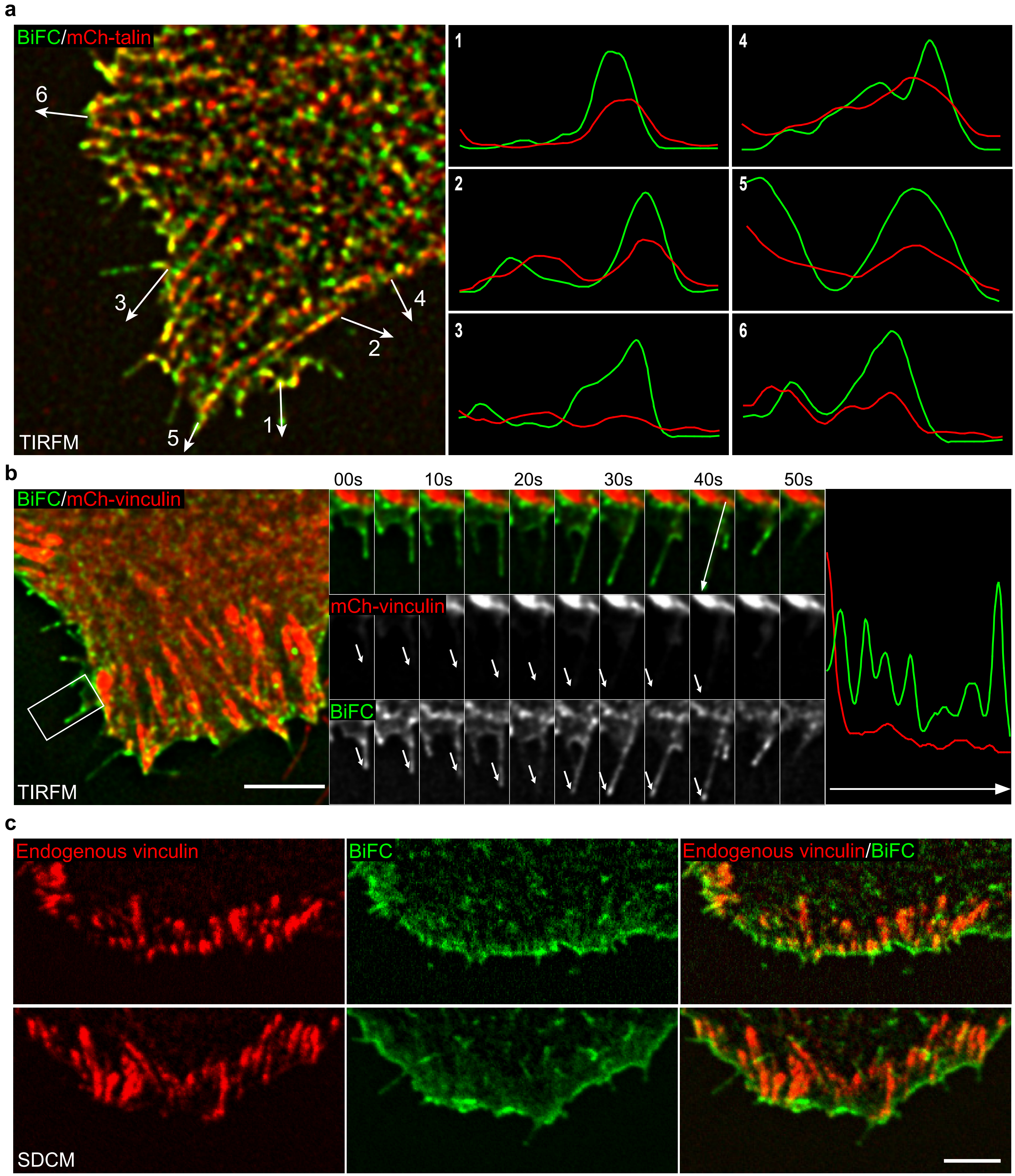

Supplementary Figure 3. BiFC colocalizes with talin but not vinculin. U2-OS cells expressing VN-RIAM, integrin α_{IIb}-VCβ_3 and mCherry-talin were plated on fibrinogen for 2 h before imaging with TIRFM. Line scans were performed along multiple filopodia and displayed. Scale bar: 5 µm. (b) mCherry-vinculin was localized distal to the BiFC. U2-OS cells expressing VN-RIAM, integrin α_{IIb}-VCβ_3 and mCherry-vinculin were plated on fibrinogen for 2 h before imaging with time-lapse TIRFM. A segment of the cell edge is enlarged and displayed as a movie montage at 5 sec intervals. The arrows show the BiFC enrichment at the tip of the growing filopodium. The line scan was drawn along the extended filopodium as highlighted in the box at 50 sec. Scale bar: 5 µm. (c) U2-OS cells expressing VN-RIAM and integrin α_{IIb}-VCβ_3 were plated on fibrinogen for 2 h. The cells were fixed and stained for endogenous vinculin and imaged by spinning disk confocal microcopy (SDCM). Two representative cells are shown. Scale bar: 5 µm.

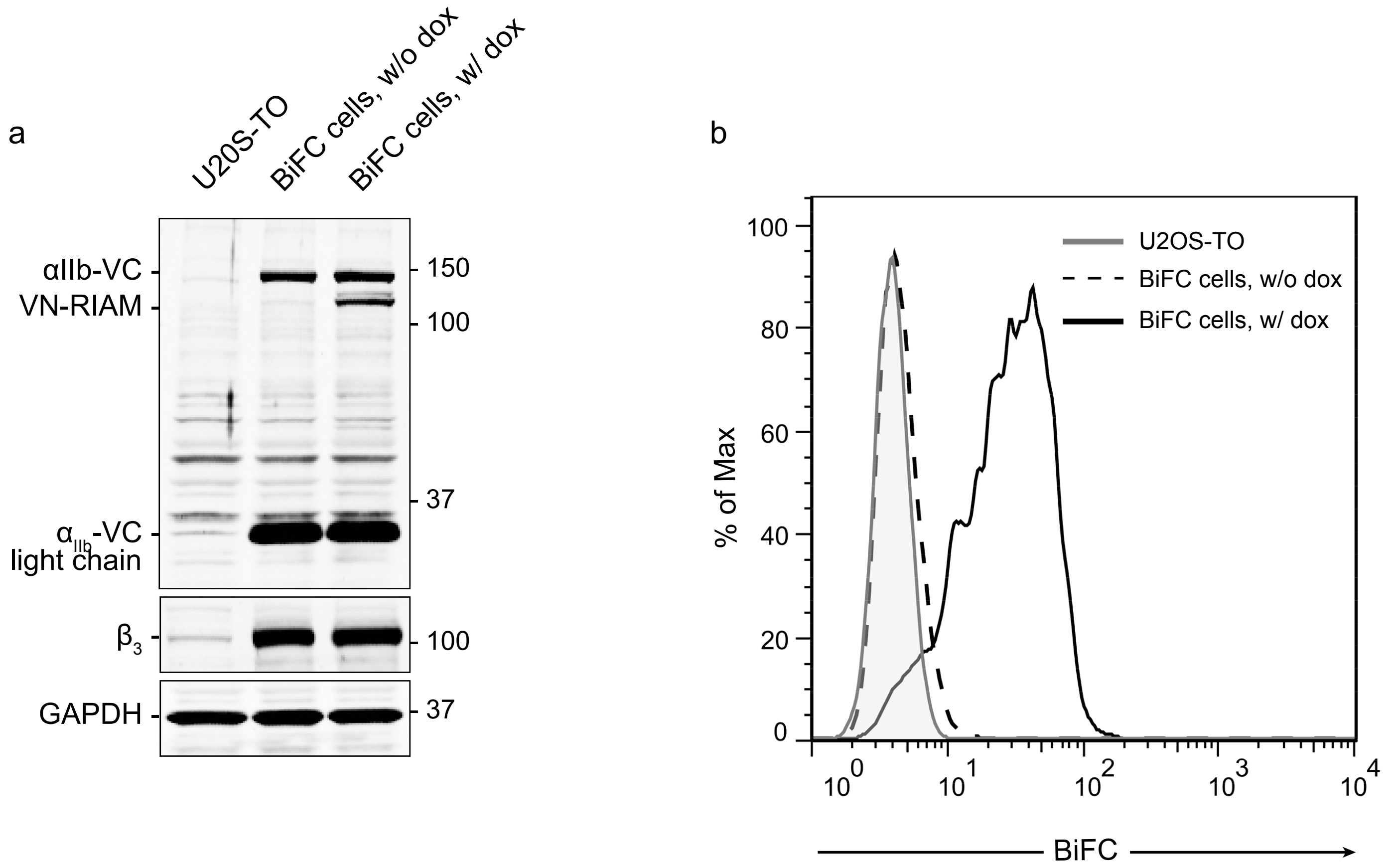

Supplementary Figure 4. Characterization of a U2-OS cell clone with tunable BiFC. U2-OS Tet-On cells exhibited constitutive expression of integrin α_{IIb}-VCβ_3 and inducible expression of VN-RIAM in presence of doxycycline. (a) Cell lysates were analyzed by Western blot with polyclonal anti-EGFP (cross-reacts with both VN and VC) and anti-integrin β_3 antibodies. GAPDH was used as a loading control. (b) Cells were harvested, fixed, and BiFC fluorescence intensity was analyzed by flow cytometry.

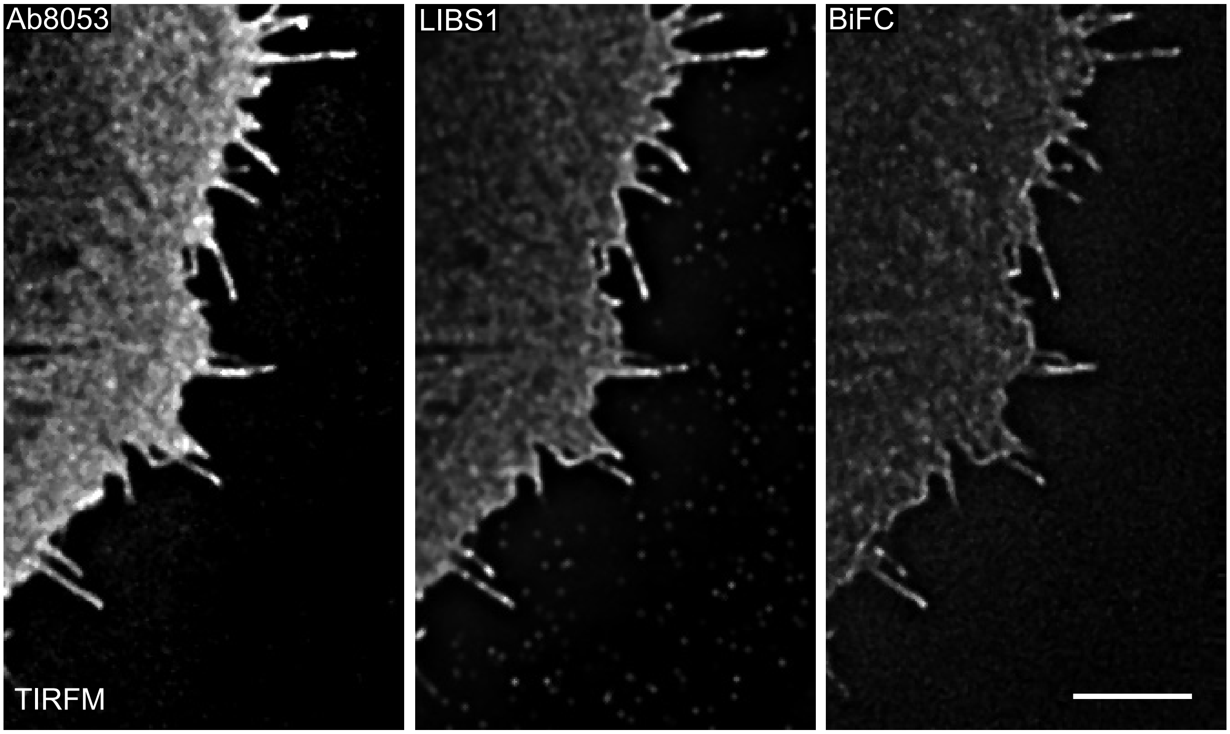

Supplementary Figure 5. U2-OS cells expressing VN-RIAM and integrin α_{IIb}-VCβ_3 were plated on fibrinogen for 2 h, fixed, stained with anti-LIBS1 (activated and ligand-bound $\alpha_{IIb}\beta_3$ integrin) and Rb8053 (total $\alpha_{IIb}\beta_3$ integrin) antibodies and imaged with TIRFM. Scale bar: 5 μm.

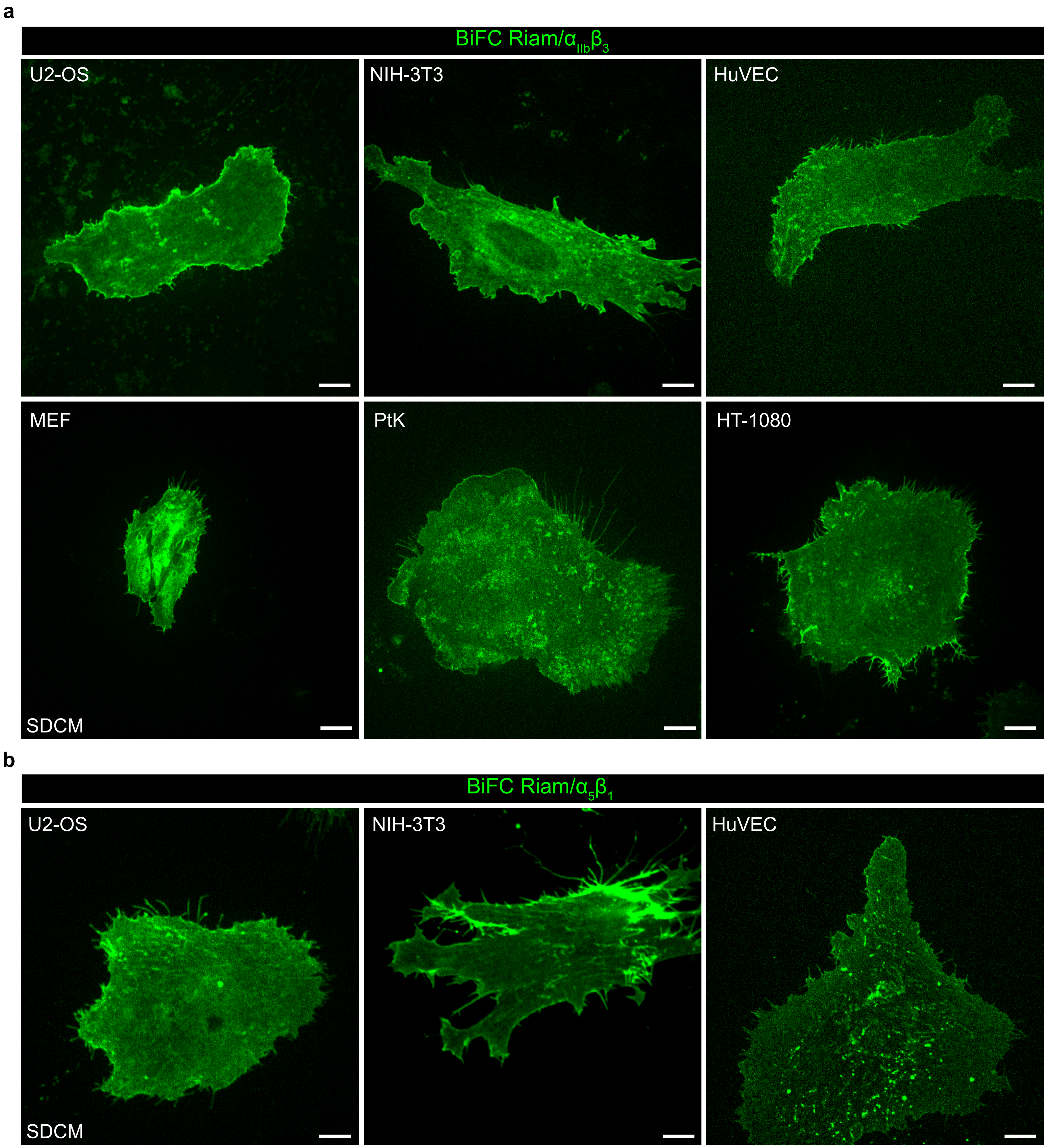

Supplementary Figure 6. The MIT complex forms in multiple cell types and with different integrins. (a) U2-OS, NIH-3T3, HuVEC, MEF, PtK1, and HT-1080 cells expressing VN-RIAM and integrin α_{IIb}-VCβ_3 were plated on fibrinogen for 2 h and imaged for BiFC signal by spinning disk confocal microscopy (SDCM). (b) U2-OS, NIH-3T3, and HuVEC expressing VN-RIAM and integrin α_5-VCβ_1 were plated on fibronectin for 2 h and imaged for BiFC signal by spinning disk confocal microscopy (SDCM). Scale bar: 10 μm.

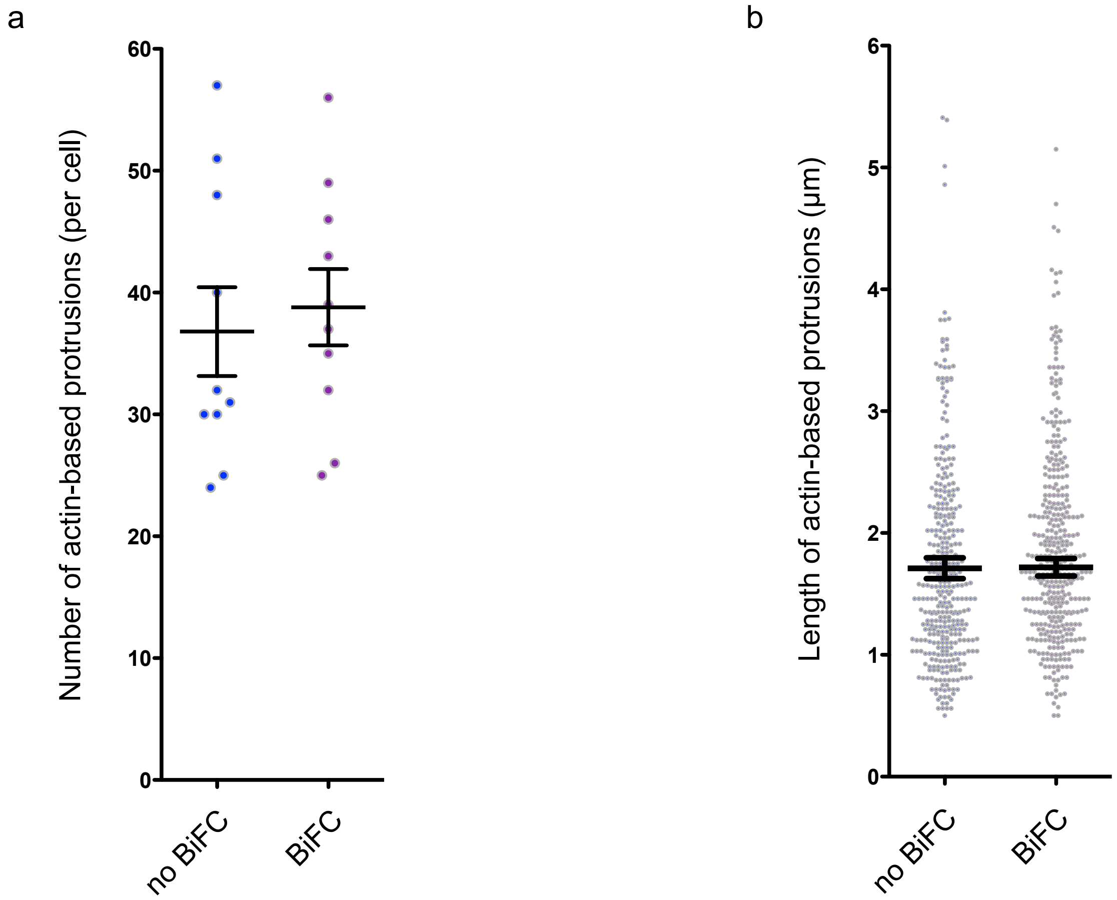

Supplementary Figure 7. BiFC does not enforce the formation of actin-based protrusions extending from the cell edge. U2-OS cells expressing either integrin α_{IIb}-VCβ_3 alone (no BiFC) or α_{IIb}-VCβ_3 and VN-RIAM (BiFC) were plated on fibrinogen for 2 h, fixed and stained with phalloidin before imaging by spinning-disk confocal microscopy. Volocity was used to draw line across actin-based protrusions extending from the cell edge, from the tip to the cell body. The number of such lines measures the number of actin-protrusions per cell and the lengths of the lines measures their lengths. All protrusions larger than a 0.5 µm cutoff were considered for analysis. (a) The numbers of actin-based protrusions from 10 randomly picked cells were quantified. Data are expressed as number per cell. P=0.68, two-tailed T-test. (b) The length of actin-based protrusions from cells analyzed in (a) was measured. Without BiFC, n=366 protrusions; with BiFC, n=387 protrusions. P=0.097, two-tailed T-test. The error bars display SEM.

Figure 4b

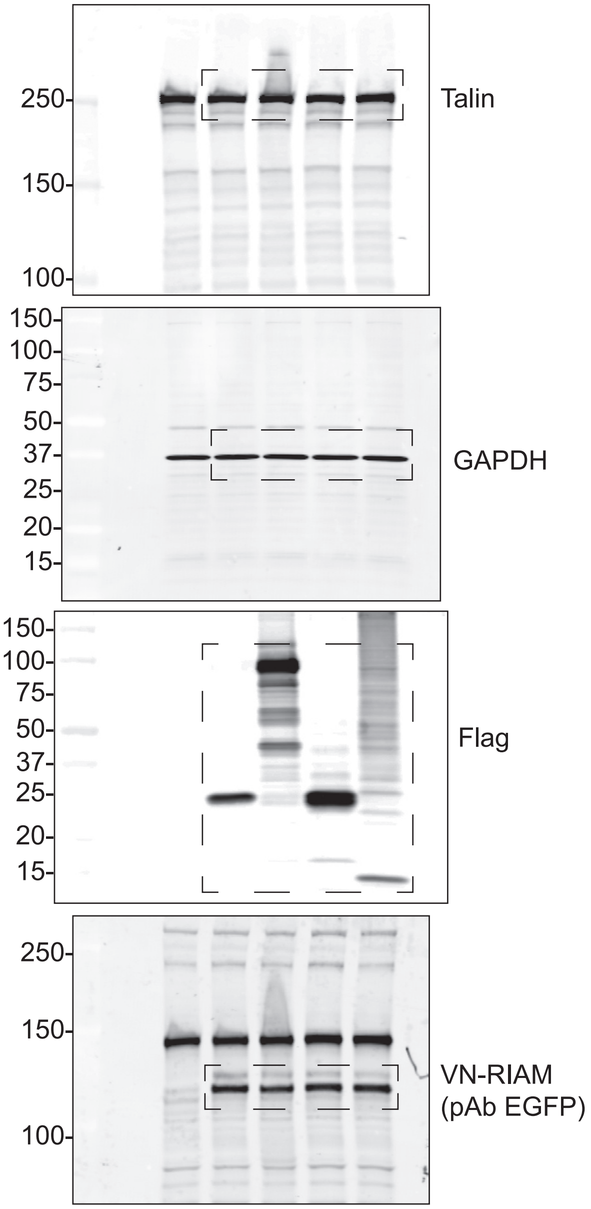

Supp Figure 4a

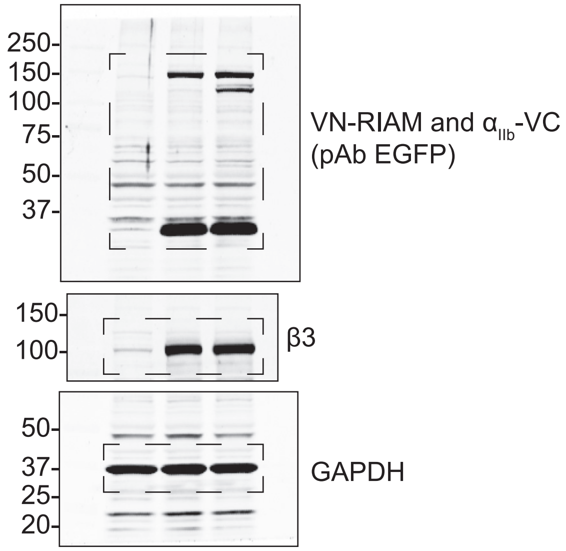

Figure 6c

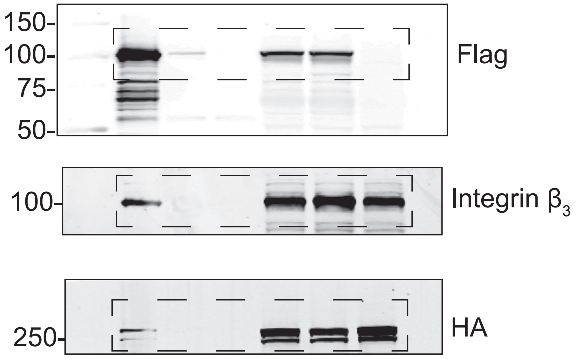

Figure 8a

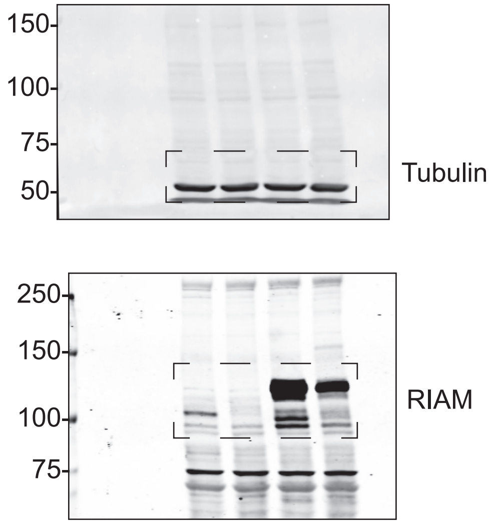

Supplementary Figure 8. Uncropped immunoblots comprising panels in Figures.
